# Quantifying Olfactory and Alveolar Deposition of Ultrafine Particles Using Multiscale Modeling: Implications for Brain Exposure

**DOI:** 10.1021/acs.est.6c05750

**Published:** 2026-06-25

**Authors:** Karine Sartelet, Lya Lugon, Soo-Jin Park, François Gaie-Levrel

**Affiliations:** † 521050 CEREA, ENPC, Institut Polytechnique de Paris, EDF R&D, IPSL, 77 455 Marne la Vallée, France; ‡ AIRPARIF, The Air Quality Observatory for the Paris Region, 77 004 Paris, France

**Keywords:** lung deposition surface area, particle size distribution, traffic, wood heating, particle translocation, particle number, hygroscopic growth, children

## Abstract

Atmospheric particle exposure is commonly characterized using mass-based metrics that inadequately capture particle surface area, respiratory deposition, and direct brain delivery pathways. Ultrafine particles (UFPs) contribute marginally to PM_2.5_ mass yet account for a disproportionate fraction of deposited surface area in both the lungs and the nasal olfactory region, a critical gateway for direct particle translocation to the brain. Using multiscale atmospheric modeling from continental to street level, evaluated against NO_2_, particulate mass, chemical composition, and size-resolved particle observations, we quantify particle deposition through lung-deposited surface area (LDSA) and olfactory deposition, explicitly accounting for hygroscopic growth. UFPs account for more than one-third of alveolar surface deposition in urban environments, and particles smaller than 400 nm dominate alveolar deposition despite their marginal contribution to PM_2.5_ mass. Decoupling between PM_2.5_ and deposited surface area persists across cities, source contributions, and population groups, with children experiencing approximately 3-fold higher alveolar doses than adults. Residential wood heating dominates winter PM_2.5_ and alveolar deposition, whereas traffic controls particle number and olfactory deposition. Critically, under the tested translocation assumptions, olfactory deposition of UFPs is estimated to exceed blood-borne translocation to the brain by several hundred-fold, supporting the relevance of neuronal pathways for brain-relevant particle delivery and consistent with recent observations of black carbon in the human olfactory bulb.

## Graphical abstract

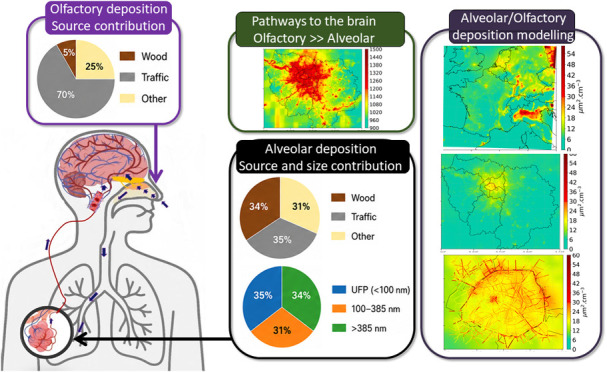

## 1 Introduction

Ultrafine particles (UFPs, diameter lower than 100 nm) are increasingly recognized as a distinct and health-relevant class of air pollutants because of their high number concentrations, deep lung penetration, and ability to cross biological barriers.^1,2^ Despite these properties, UFPs are not included in mass-based air quality regulations such as PM_2.5_ or PM_10_ owing to their negligible contribution to total particle mass. Their importance is nevertheless recognized in the new EU Air Quality Directive (2024/2881/CE), which calls for the quantification of UFP using particle number concentration (PNC), a metric better suited than mass for this size range.

Epidemiological evidence on the health effects of UFP exposure remains limited and heterogeneous, partly due to the scarcity of long-term measurements and spatially resolved exposure data.^3^ Associations with mortality and hospital admissions are less consistent for UFPs than for PM_2.5_ or particle surface area metrics.^4,5^ Nonetheless, several distinct health outcomes have been reported, including delayed cardiovascular and respiratory effects, particularly in children.^5–8^ Emerging evidence further links air pollution exposure to neurological outcomes,^9^ with a potential preponderant role of UFPs.^10–12^

Toxicological studies consistently indicate that particle surface area is a more significant predictor of biological responses than mass-based metrics. Measures of particle surface concentration have been linked to inflammatory blood markers Surface area is widely recognized as the most relevant dose metric for acute nanoparticle toxicity in the lung.^13,14^

While toxicological studies consistently identify particle surface area as a key driver of biological responses, ambient surface area alone does not represent the dose delivered to respiratory tissues because deposition efficiency depends strongly on particle size and airway geometry. Lung-deposited surface area (LDSA) addresses this limitation by combining particle surface area with size-dependent deposition fractions, thereby providing an estimate of the surface area actually delivered to specific regions of the respiratory tract.^15^ LDSA is particularly relevant for UFPs, which deposit efficiently in the extrathoracic (ET), tracheobronchial (TB), and alveolar (AL) regions.^2,16^ Deposition in the alveolar region (LDSA_AL_) is of particular interest,^15^ as it facilitates rapid translocation into the bloodstream owing to the thin epithelial barrier, large available surface area, and relatively low clearance rates.^1,17^ As a result, UFPs may cross the air-blood barrier and become systemically available, enabling distribution to peripheral organs such as the liver, kidneys, and brain.^18–20^

Beyond pulmonary deposition, particle size also influences deposition pathways in the upper respiratory tract. UFPs can directly enter the brain by extrathoracic deposition-derived neuronal pathways, such as the olfactory and trigeminal pathways.^1,21,22^ Due to their small size, UFPs deposited in the olfactory epithelium may be transported along the olfactory nerve.^17,23^ Recent human evidence has demonstrated the presence of black carbon particles, predominantly in the ultrafine size range, in the brain, with particularly high concentrations observed in the olfactory bulb.^24^ This is consistent with recent evidence on nose-to-brain particle transport and with experimental studies showing that traffic-related UFPs can affect human olfactory mucosa cells.^25,26^ The abundance of α-synuclein in this region supports a link between olfactory deposition of UFPs and neurological effects,^10^ consistent with its key role in neurodegenerative diseases^27,28^ and with recent cohort evidence linking long-term UFP exposure to dementia.^29^ We therefore estimate the olfactory lung-deposited surface area (LDSA_Olf_) to quantify this pathway and compare it with alveolar translocation.

High alveolar LDSA values are observed in urban environments owing to high particle number concentrations and small particle sizes, with strong intraurban variability driven by traffic emissions.^30–36^ Such variability has motivated recent high-resolution modeling of UFP and particle number concentrations using mobile monitoring, land-use regression, machine learning, and deterministic atmospheric modeling.^3,37–40^ Mobile monitoring has also provided valuable urban-scale maps of LDSA.^34–36^ However, such measurements typically reflect ambient or instrument-conditioned particle size distributions and do not explicitly account for hygroscopic growth during inhalation, which can alter deposited surface area and regional deposition fractions. Because LDSA is highly sensitive to particle size, its estimation requires explicit representation of aerosol microphysics and hygroscopic growth. Moreover, high alveolar LDSA does not necessarily coincide with high PM_2.5_ mass,^15,34^ and the relative contributions of particle size and emission sources to region-specific LDSA remain insufficiently quantified, particularly across spatial scales and deposition regions. While alveolar LDSA has been increasingly investigated in recent years, information on olfactory LDSA remains extremely limited, with no spatially resolved assessments available. To date, no study has provided a spatially resolved multiscale quantification of ultrafine particle deposited surface area in both the alveolar and olfactory regions while explicitly accounting for aerosol microphysics. As a result, the spatial variability of these pathways, their age dependence, and their relative importance for potential brain exposure remain largely unknown. In this study, we develop a multiscale atmospheric modeling framework to quantify olfactory and alveolar lung-deposited surface area (LDSA_Olf_ and LDSA_AL_) from regional to street scales, accounting for particle size, aerosol microphysics, and hygroscopic growth. We then examine their variability with age, focusing on 3-year-old children, a vulnerable population because of higher breathing rates relative to body size and increased susceptibility during early development, and their dependence on major emission sources, including traffic and residential wood combustion. This framework enables a quantitative comparison of olfactory and alveolar pathways and provides new insights into health-relevant ultrafine particle exposure.

## 2 Methodology

### 2.1 Multiscale Simulations

Air-quality simulations are carried out using the CHIMERE/MUNICH/SSH-aerosol modeling chain,^41^ with the same configuration as Lugon et al.^42^ Spatial and temporal variability are determined by emission inventories, meteorology and dispersion, boundary conditions, chemistry, aerosol microphysics, and deposition. CHIMERE^43^ is coupled with MUNICH^44^ and the SSH-aerosol module (version 2.0)^45^, which describes aerosol processes such as nucleation, coagulation, and condensation/evaporation. The particle size distributions are represented in 10 size bins, ranging from 0.01 to 10 μm in diameter. A three-month period between 1 December 2020 and 28 February 2021 is simulated. This period coincides with an Airparif measurement campaign in Île-de-France, during which ultrafine particles were also monitored.

To capture the spatial variability of concentrations, regional and urban background concentrations are simulated on nested grids and combined with street-network modeling driven by hourly traffic emissions and meteorological conditions. The simulations over Île-de-France are performed at a resolution of 1 km × 1 km. Larger-scale simulations provide boundary conditions through a nested-domain approach. Three nested domains are used: (i) the outer FRA9 domain, which covers France and part of Western Europe (1476 km × 1566 km) at 9 × 9 km^2^ resolution; (ii) the intermediate IDF3 domain, centered on the Île-de-France region (495 km × 513 km) at 3 × 3 km^2^ resolution; and (iii) the innermost IDF1 domain, focusing on Île-de-France at 1 × 1 km^2^ resolution (Figure 1). The finest-resolution street-scale domain is suitable for high-resolution outdoor exposure assessment, including address or street-level contrasts, whereas the coarser domains should be interpreted as background exposure fields because they do not resolve near-road UFP gradients.

**Figure 1:**
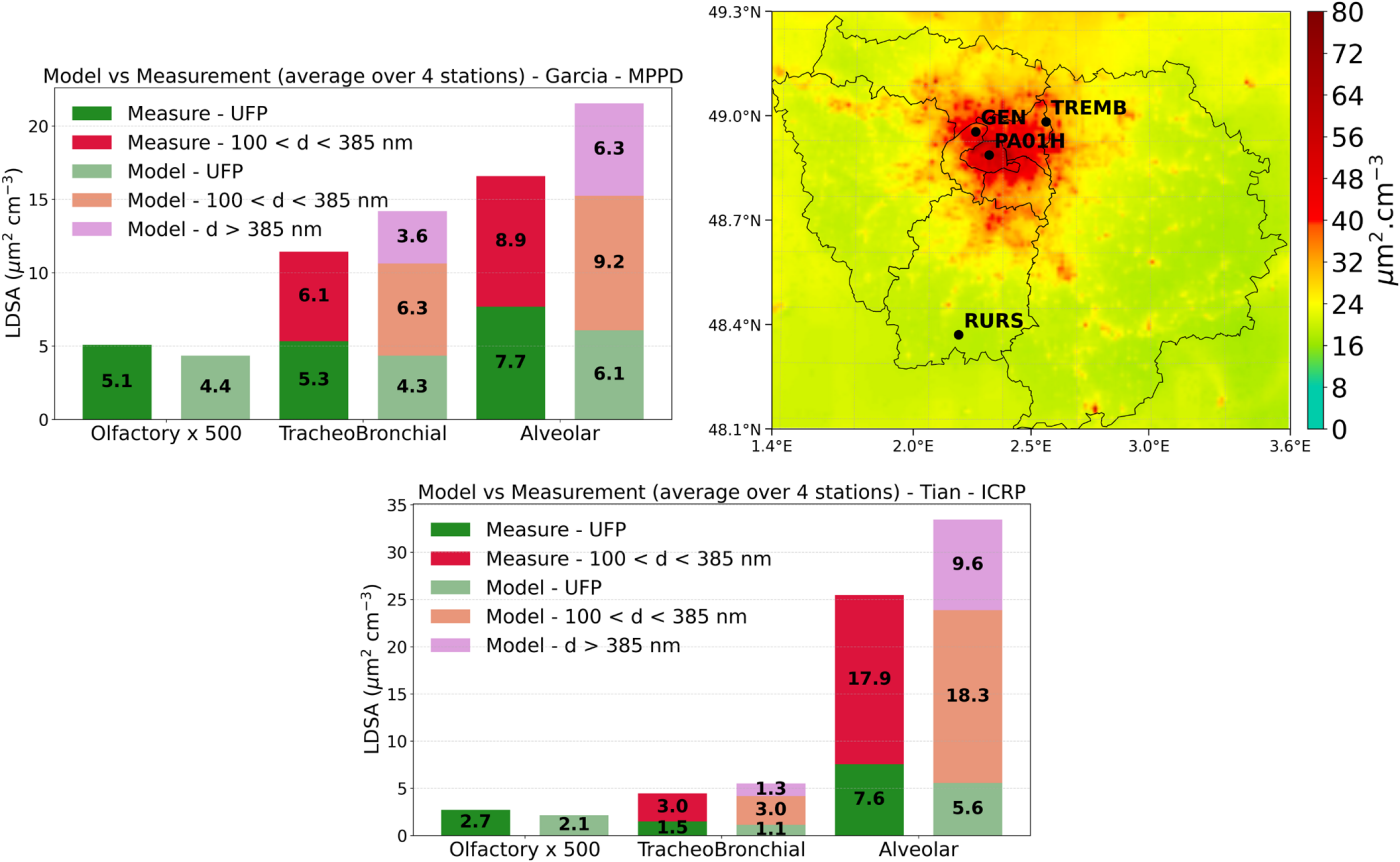
Maps of LDSA over Île-de-France with the location of the measurement stations (top right panel), calculated using the MPPD deposition fractions. Left panels: observed and simulated “dry” LDSA averaged over 4 measurement stations for the simulation period (December 2020 to February 2021, left panel). The LDSA is computed using the MPPD and Garcia et al.^21^ deposition fraction in the top left panel, and using ICRP and Tian et al.^47^ deposition fraction in the bottom panel.

### 2.2 Calculation of LDSA

In each region *r* of the respiratory tract (TB and AL), LDSA is inferred from the number size distribution of particles and the particle lung deposition function *F*_*r*_ estimated with the Multiple-Path Particle Dosimetry model (MPPD v3.04)^46^ for a healthy male adult at rest and for a 3-year-old child.

1 $$\mathrm{LDS}A_{r}=\sum_{i}N_{i}\pi d_{p,i}^{2}F_{r,i}$$

where *N*_*i*_ is the number of particles of mean aerodynamic diameter *d*_p,*i*_, *F*_*r*,*i*_ is the deposition fraction in the respiratory tract region *r* for particles of diameter *d*_p,*i*_.

For particles of aerodynamic diameters lower than 100 nm, the LDSA in the olfactory region is estimated from^21^

2 $$\mathrm{LDS}A_{\mathrm{Olf}}=\sum_{i}N_{i}\pi d_{p,i}^{2}F_{\mathrm{nasal},i}F_{0,i}$$

where *F*_nasal,*i*_ and *F*_0,*i*_ are respectively the fraction of the particles depositing in the nasal region and the fraction of those particles that subsequently deposit in the olfactory region. They are estimated using the curved fitted from computational fluid dynamics simulations by.^21^ As a sensitivity study, the nasal deposition fraction is also estimated using the parametrization of.^47^ Because olfactory deposition in the 10–100 nm particle size range scales with olfactory surface area,^21^ the olfactory LDSA computed using eq 2 is scaled by a factor of 0.64 for a 3-year-old child (see Supporting Information).

#### 2.2.1 Size-Resolved and Composition-Weighted LDSA

LDSA concentrations are calculated using deposition fractions from MPPD based on the particle diameter at 99.5% relative humidity, representative of conditions in the respiratory tract. Inorganic and organic aerosol compounds absorb substantial amounts of water at high relative humidity, which markedly increases particle size and thus the calculated LDSA. Because water is nontoxic, this artificial enhancement does not represent the actual particle burden on the human body. The LDSA concentrations derived from humidified particles are therefore corrected by the dry-to-wet mass ratio, which removes the water contribution to only keep deposition of particulate matter components of toxicological relevance.

To further enhance the relevance of the LDSA as a health-related exposure metric, we propose adjusting it according to the intrinsic toxicities of the dominant particle compounds. This weighting scheme is consistent with recent multiassay studies linking chemical composition and source contributions to oxidative potential.^48,49^ The toxicity weighted LDSA (LDSA^tox^) is calculated by weighting the LDSA by the mass of the particle compound category using the weights detailed in the Supporting Information.

#### 2.2.2 Systemic and Brain Dose Estimation

We distinguish between the systemic pathway, where particles translocate from the lung into the bloodstream and are then distributed throughout the body (including a small fraction reaching the brain across the blood-brain barrier), and the olfactory direct pathway, where particles deposited in the nasal olfactory region can bypass the circulation and enter the brain directly via neuronal transport. We consider only alveolar translocation as a source of systemic exposure, while tracheobronchial particles are assumed to be cleared locally or dissolved into blood. An intact alveolar epithelial barrier is assumed with a translocation rate of 4.2 × 10^–4^ h^–1^ as measured in vitro for diesel exhaust particles across alveolar epithelial cells.^50^ The blood-to-brain translocation fraction, across the very tight blood-brain barrier is very uncertain, and likely to be low,^51^ even for engineered nanoparticles.^52,53^ Here, a fixed fraction *f*_blood → brain_ = 0.001. For translocated to the olfactory bulb with studies reporting values of transported particles ranging between 9 and 20% for rodents,^1,17,54^ and for soluble compounds in humans.^55^ Hence, we assume a fraction *f*_Olf → bulb_ = 0.15.

## 3 Results

### 3.1 Model Evaluation

The particle size distribution was monitored using a mobility particle size spectrometer (MPSS) for particles of diameters below 385 nm at four stations: Les Halles (PA01H), which is an urban background station located in the center of Paris, Bois-Herpin (RURS), a rural station south of Paris, Genevilliers (GEN) an urban station and Tremblay-en-France (TREMB), a suburban station in the Île-de-France region.^56^ The observed LDSA values are inferred from the MPSS measurements using the same methodology as used for the modeling. Because MPSS diameters correspond to dry particle mobility diameters, the impact of humidity on particle diameter, growth and LDSA is not considered and dry LDSA are compared.

The comparisons of modeled and observed dry LDSA averaged over 4 measurement stations are presented in Figure 1. The LDSAs are on the same order of magnitude as those presented over different European stations using a similar methodology.^16^ For example, at the urban background site in central Paris (PA01H), the simulated LDSA_*AL*_ is approximately 21 μm^2^ cm^–3^, well within the range reported for European cities (12–40 μm^2^ cm^–3^) by Liu et al.^16^ The daily deposited surface area in the olfactory region is estimated at approximately 7.1 × 10^4^ μm^2^. This value is comparable to olfactory deposition reported for indoor environments by^57^ (3.6 × 10^4^ to 2.8 × 10^5^ μm^2^, depending on exposure scenario), and is about a factor 2 to 5 higher than estimates for suburban outdoor conditions reported by^58^ (1.5–3 × 10^4^ μm^2^). Outside Paris, at the rural RURS site, daily olfactory deposition is substantially lower (8.4 × 10^3^ μm^2^), lower than these suburban estimates.

For model to measurement statistics, although no criteria exists for LDSA, following the criteria defined for PM_2.5_ (based on normalized mean bias, error, correlation and the fraction of predictions within a factor of 2), the modeled LDSA compared very well to the LDSA inferred from the 4 measurement stations (see statistics in the Supporting Information). As shown in the scatter plots and temporal evolution presented in the Supporting Information, the model shows good overall agreement with observations across stations, capturing both the magnitude and temporal variability of LDSA as well as the main features of the particle number size distributions.

The number size distribution up to 385 nm is evaluated using MPSS measurements, while larger particles are assessed using complementary observations, including PM_2.5_, PM_10_, and aerosol chemical composition. Together, these comparisons support the physically coherent full size distribution provided by the deterministic modeling framework over the 10 nm to 10 μm range.

### 3.2 Physical Drivers: Humidity and Size Effects

Following the evaluation, we examine the physical processes controlling LDSA. In particular, hygroscopic growth and particle size strongly influence deposition efficiency and thus the resulting surface dose.

#### 3.2.1 Influence of Humidity

As particles enter the body where the relative humidity can reach 99%–100%,^59,60^ they may grow and their diameter may increase, because of the water absorption by inorganic and hydrophilic organic compounds of particles. Considering humidity when calculating LDSA leads to a decrease of LDSA for ultrafine particles and for particles smaller than 400 nm, because deposition velocities tend to decrease with increasing diameter below 400 nm. In contrast, the deposition of particles larger than 400 nm tends to increase, since deposition velocities are generally at a minimum in the 400 nm to 2 μm size range. Figure 2 compares the wet and dry LDSA simulated in central Paris (PA01H). For ultrafine particles, humidity leads to a decrease of the total LDSA by almost 60%. As shown in Figure 2, the decrease of LDSA because of humidity is more pronounced at the RURS than at PA01H station, i.e., outside of urban areas and further away from sources, as particles tend to be more aged and contain more hydrophilic compounds. Humidity leads to a decrease of LDSA_AL_ and LDSA_Olf_ relative to the dry-particle calculation that can reach 60% and 90% respectively in rural areas in northern Europe (see Supporting Information). On average, the impact of humidity in the streets of Paris is similar although slightly lower than in the urban background, it leads to a decrease of 35% and 40% in the streets and in the urban background respectively for LDSA_*AL*_ and of 35% and 46% for LDSA_Olf_. The impact on LDSA_Olf_ is larger than the impact on LDSA_AL_, as the deposition velocities of UFP consistently decreases with particles diameters in the nasal region in opposite to the AL region, where the decrease only concerns particles of diameters larger than about 20 nm. Furthermore, only ultrafine particles contribute to LDSA_Olf_, whereas the decrease of LDSA_AL_ because of ultrafine particles is compensated by the increase of deposition of particles larger than 400 nm.

**Figure 2:**
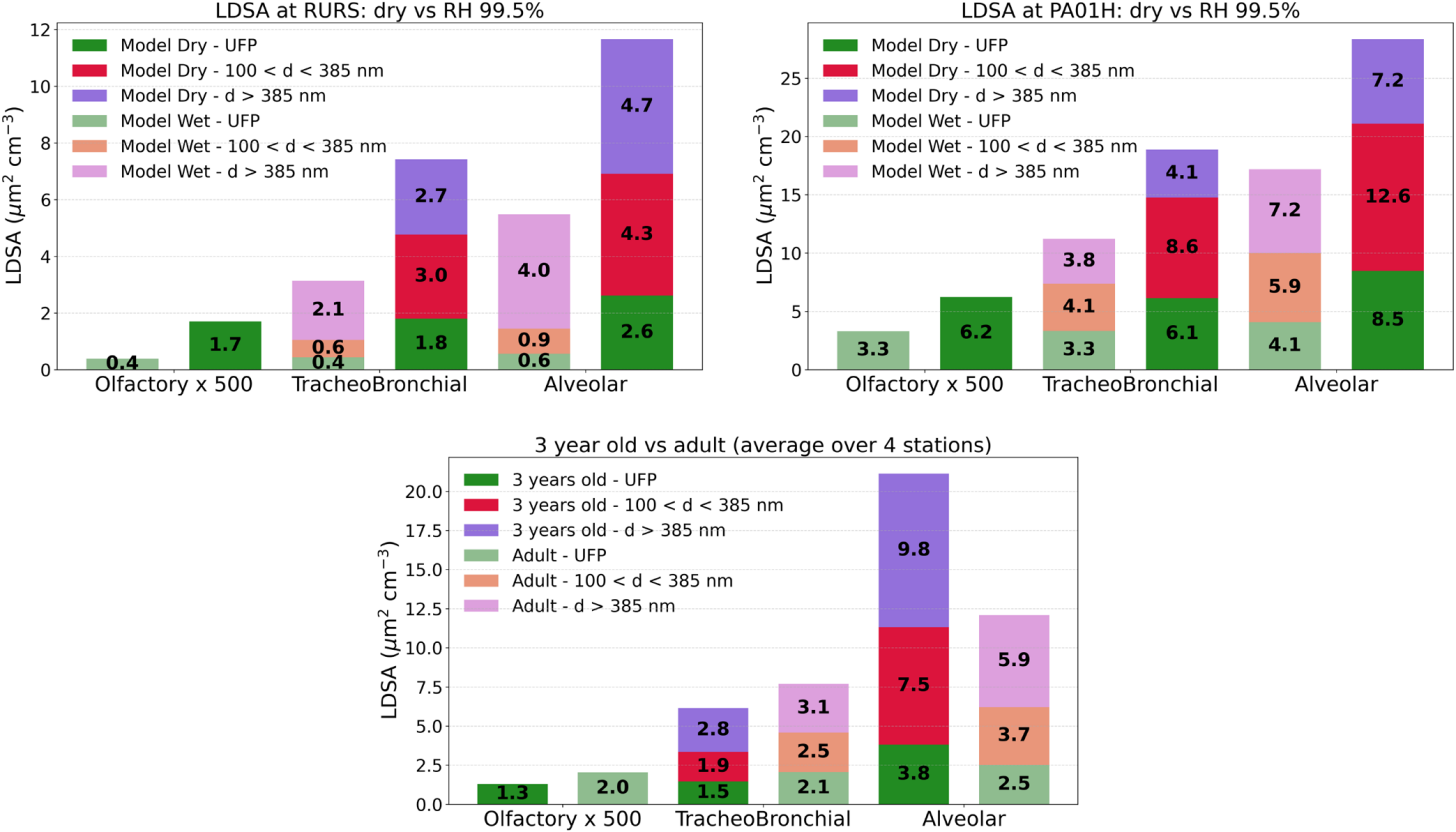
Top panels: simulated LDSA at rural (RURS, top left panel) and urban station (PA01H, middle right panel); simulations with and without taking into account the water absorbed by particles in the calculation of the deposition fractions are compared. Bottom panel: simulated three-year-old child and adult LDSA over 4 locations (GEN, PA01H, RURS, TREMB) (left panel); water absorbed by particles is taken into account.

#### 3.2.2 Particle Size Contributions to LDSA_AL_

Measurements across all winter monitoring sites show that UFPs dominate particle number concentrations (PNC), contributing between 75% and 86% depending on location. The highest contributions are observed at urban and traffic-influenced sites, such as Gennevilliers (GEN, 86.4%), Paris center (PA01H, 83.7%) and Tremblay-en-France (TREMB, 84.3%), whereas the rural background site (RURS) exhibits a lower contribution (75.3%), consistent with its greater distance from emission sources. Model simulations reproduce these patterns well (Figure 3), confirming a strong dominance of UFPs over cities and along major transportation corridors. Across Western Europe, UFPs account on average for about 74% of PNC, with values ranging from 23% to 93%. Over Paris, this contribution increases to approximately 81%. Particles smaller than 400 nm account for nearly all (95–100%) of the simulated PNC, highlighting the overwhelming role of the ultrafine size range.

**Figure 3:**
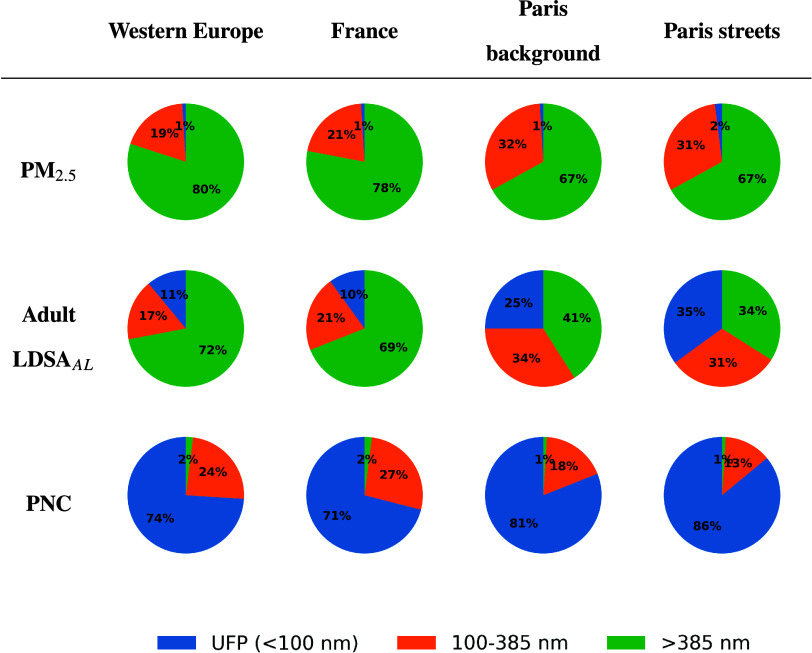
Average contribution of ultrafine particles (in blue) and of particles of diameters between 100 and 385 nm (in orange) and larger particles (in green) to PM_2.5_, LDSA_*AL*_ (adult), PNC over Western Europe, France, Paris background and Paris streets.

UFPs contribute very little to PM_2.5_ mass concentrations, typically only 1–2% across all spatial domains, but UFPs represent a substantial fraction of LDSA_AL_ (Figure 3). At regional scales, UFPs contribute on average 10–11% of LDSA_AL_ over Western Europe and France. This contribution increases markedly in urban environments, reaching 25–35% over Paris and at the street level within the city. Similar UFP contributions are simulated for other major European cities (Supporting Information), indicating that this pattern is not specific to Paris but characteristic of dense urban settings.

Particles in the 100–385 nm size range also make a substantial contribution to LDSA_*AL*_, in some cases comparable to or exceeding that of UFPs. Over Western Europe and France, these particles account for about 21–28% of LDSA_AL_, increasing to 31–34% over Paris. Unlike UFPs, their contribution to PM_2.5_ mass is of similar magnitude, reflecting their larger size and mass content.

### 3.3 Spatial Variations and Correlations between Indicators

At large spatial scales, the spatial distribution of LDSA_AL_ (Figure 4) broadly follows that of PM_2.5_, with relatively smooth regional patterns and marked contrasts between urban and rural areas. Across Western Europe, LDSA_AL_ ranges from near-zero values over oceanic regions to elevated concentrations exceeding 25 μm^2^ cm^–3^ in densely populated areas and in northern Italy’s Po Valley. Over Île-de-France, there is a clear urban-rural gradient, with higher LDSA_AL_ concentrations in the Paris metropolitan area (20–30 μm^2^ cm^–3^) that decrease to background levels of about 5–15 μm^2^ cm^–3^ in surrounding rural regions. In contrast, olfactory-deposited surface area (LDSA_Olf_) displays much stronger spatial heterogeneity. Elevated LDSA_Olf_ concentrations are tightly confined to urban environments and vary sharply over short distances. At the city scale, LDSA_Olf_ closely follows the street network, with distinct linear features along major roadways in Paris, indicating pronounced local contrasts in brain-relevant exposure.

**Figure 4:**
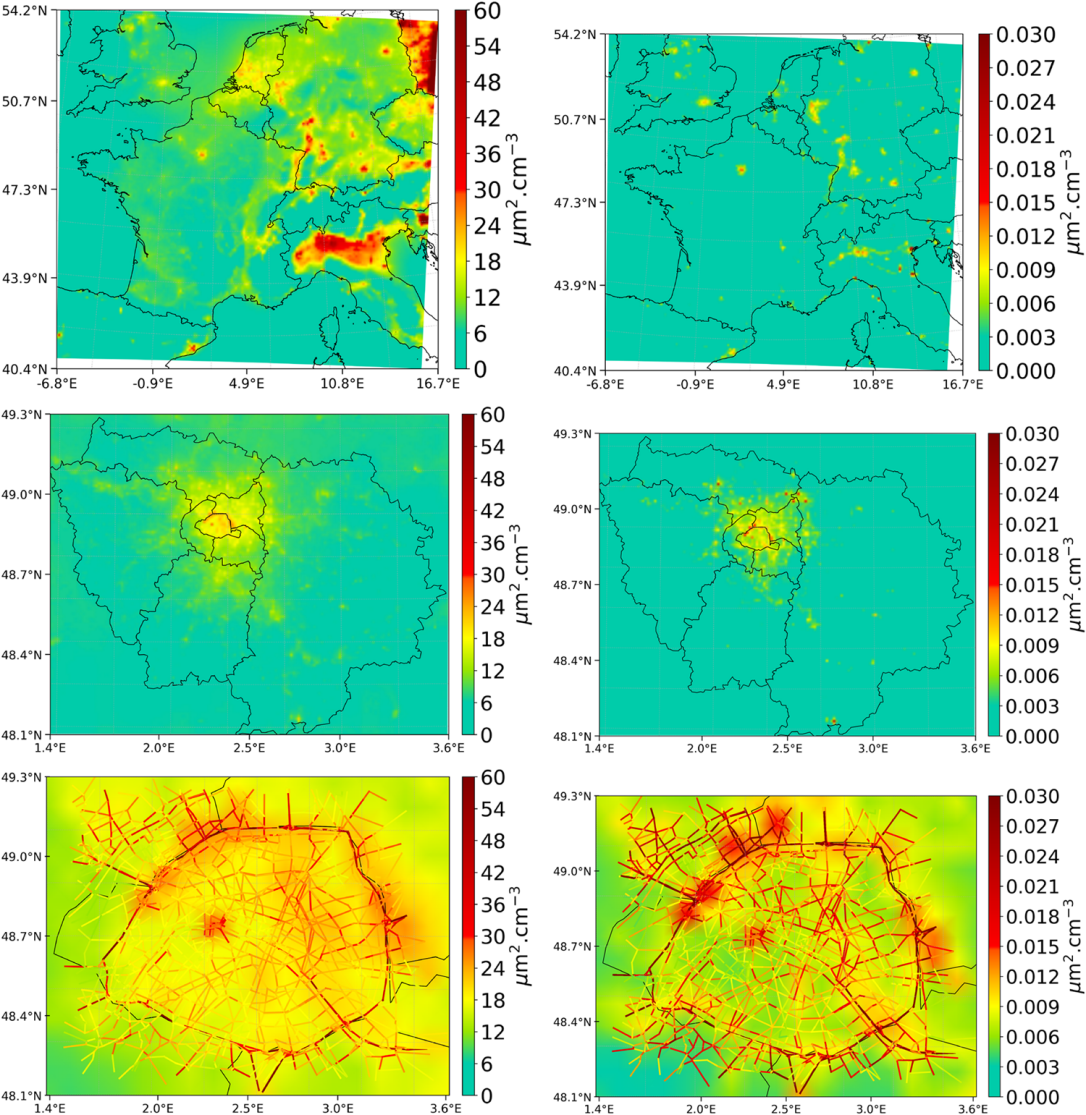
Maps of LDSA_AL_ (left columns) and LDSA_Olf_ (right columns) over France (top panels), Île-de-France (second line panels) and Paris (third line panels).

The UFP contribution to LDSA_AL_ also shows strong spatial variability across the studied domains (see Supporting Information). The contrast between Western Europe, Paris background, and Paris street environments clearly demonstrates the enhancement of the UFP contribution in cities and in traffic-impacted areas. The street sites exhibiting the highest proportion of UFP and the lowest fraction of particles of diameters larger than 385 nm, underscoring the importance of local traffic emissions for surface-based exposure metrics.

Quantitative analyses (Supporting Information) further indicate that surface-based exposure metrics vary more strongly in space than particle mass, particularly at fine spatial scales. Accounting for particle-bound water (i.e., humid rather than dry LDSA) increases the spatial variability of LDSA metrics, because of hygroscopic growth during atmospheric aging. PM_2.5_ mass explains a substantial fraction of the spatial variability in alveolar-deposited surface area, but provides limited information on olfactory deposition. PNC offers complementary insight by better reflecting ultrafine particle-driven variability, particularly for LDSA_Olf_, yet does not fully capture the variability of either alveolar or olfactory deposited surface area.

### 3.4 Adult vs 3-Year-Old Child

Particle deposition in young children differs from adults because of differences in breathing patterns, lung morphology, and airway dimensions. As a result, children are expected to experience higher deposited particle doses for the same ambient exposure. For three-year-old children, the relative contribution of UFPs to LDSA_AL_ is similar to that of adults, although slightly lower by a few percent, consistent with differences in breathing patterns and lung morphometry.

The bottom panel of Figure 2 compares simulated lung- and olfactory-deposited surface area (LDSA) for three-year-old children and adults at four representative locations (GEN, PA01H, RURS, TREMB). Consistent with size-dependent deposition curves (see Supporting Information), LDSA in the alveolar regions is higher in three-year-old children than in adults, whereas olfactory LDSA is lower by a factor 0.64, as detailed in section 2.2. Across all spatial domains, LDSA_AL_ is higher in three-year-old children than in adults. The child-to-adult enhancement factor for LDSA_*AL*_ exhibits limited spatial variability, generally ranging between 1.7 and 1.8 (see Supporting Information). A three-year-old child’s dose per kilogram is about 1.8 times the ratio of the LDSA levels between children and adults (see Supporting Information). Hence, the body-weight-normalized dose in three-year-old children is approximately 3.2 times higher than in adults for the alveolar region, and about 1.2 times higher for the olfactory region.

### 3.5 Source Contributions

The contributions of wood heating and traffic to PM_2.5_, PNC and LDSA are quantified using the brute-force method in Figure 5. Traffic and residential wood combustion were selected for detailed analysis because they represent two dominant sources influencing particle concentrations in cities during winter conditions, with traffic dominating UFP number concentrations and residential wood combustion PM_2.5_.^42^ Over France, the contributions are quantified through sensitivity simulations in which either wood heating or traffic emissions are excluded, and then compared with baseline simulations including all sources. A similar methodology is applied at the Île-de-France scale, where simulations are performed without wood heating or traffic emissions over Île-de-France.

**Figure 5:**
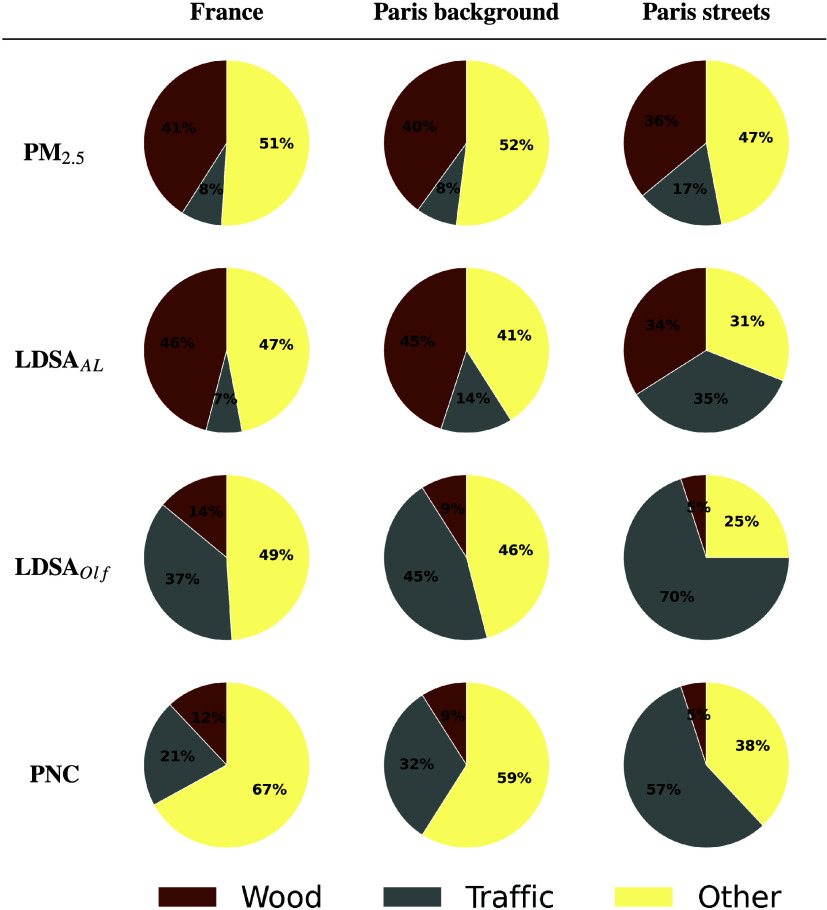
Average contribution of wood heating and road traffic to PM_2.5_, LDSA_AL_, LDSA_Olf_, PNC over France, Paris background and Paris streets. While over France the contribution is linked to nationwide wood heating and traffic, over Paris it is only from regional wood heating and traffic.

As expected,^61^ wood heating makes a very large contribution to PM_2.5_ over France (about 41% on average) and in cities, whereas the contribution from traffic remains comparatively low (around 8%). A similar picture emerges for LDSA_AL_, which is consistently dominated by wood heating across all domains (45%–46%). However, in Parisian streets the contribution of wood heating and traffic are similar (34%–35%). These results highlight that metrics linked to particle mass or deep-lung surface area are primarily driven by wood heating, although traffic becomes an equally important contributor to local LDSA_AL_ exposure levels in urban streets.

In contrast, LDSA_Olf_ and PNC both exhibit a stronger traffic influence. At the street scale, the traffic contribution increases substantially, reaching about 70% on average for LDSA_Olf_. This progressive shift indicates that deposition in the olfactory region is particularly sensitive to ultrafine, near-road aerosols. The contribution of traffic to PNC and LDSA_Olf_ becomes increasingly important when going down the scales from France to the Paris background and finally to the street scale (Paris streets). This trend is partly explained by the differences in spatial resolution: over France, concentrations represent averages over 9 km × 9 km grid cells, whereas in Paris the representativeness improves to 1 km × 1 km at the urban background, and to the street level in the Paris street domain.

### 3.6 Pathways to the Brain

Ultrafine particles may reach the brain through two main pathways: direct neuronal transport following nasal deposition, and indirect translocation via the bloodstream after alveolar deposition. Quantifying the relative importance of these pathways is essential for interpreting brain-relevant particle exposure.

Accounting for translocation efficiencies, the daily dose reaching the olfactory bulb via the olfactory pathway at PA01H is estimated at 1.1 × 10^4^ μm^2^. In contrast, the dose reaching the brain via the bloodstream is much smaller, at approximately 9 μm^2^, corresponding to a difference of more than 3 orders of magnitude (factor ∼1200). A similar contrast is found at the rural RURS site, where the olfactory-route brain dose remains about 972 times larger (1.3 × 10^3^ μm^2^) than that estimated from systemic circulation (1.3 μm^2^).

Previous work by Chalvatzaki et al.^58^ reported comparable particle doses delivered to the bloodstream and to the olfactory region; however, their analysis did not explicitly account for the restrictive transfer across the blood-brain barrier. When this constraint is included, our results consistently indicate that the olfactory pathway dominates particle delivery to the brain across all spatial domains.

The predominance of the olfactory pathway is further supported by recent human evidence.^24^ reported the presence of black carbon particles throughout the brain in individuals with Alzheimer’s disease, with particularly high particle loads in the olfactory bulb and deeper brain regions, consistent with preferential particle accumulation along this route.

## 4 Sensitivity Study

The sensitivity analysis focuses on selected assumptions used to derive deposited and brain-relevant doses from the simulated aerosol fields, including deposition models, translocation fractions, and toxicity weighting. Uncertainties in emissions, particle size distributions, and hygroscopic growth may affect absolute LDSA estimates, although the simulated aerosol fields are evaluated against observations.

### 4.1 Choice of the Deposition Model

#### 4.1.1 Alveolar Deposition Fractions

For the sensitivity study on the deposition model, the deposition fractions are derived from the Human Respiratory Tract Model developed by the International Commission on Radiological Protection (ICRP)^62^ for an adult at rest. The ICRP and MPPD models are conceptually similar in that they both estimate regional deposition from particle size and breathing conditions, but they differ in complexity: ICRP provides standardized semiempirical deposition fractions, whereas MPPD allows a more detailed treatment of subject-specific physiology, solving particle transport mechanistically at each airway generation using explicit morphometric geometries, accounting for diffusion, impaction, sedimentation, and interception.^63^

In our simulations, ICRP and MPPD predict similar total intrathoracic deposited surface area when the tracheobronchial and alveolar regions are combined (39 vs 36 μm^2^ cm^–3^, see Figure 1), but differ in the partitioning between these two regions. Alveolar deposition of UFP is similar in both models, whereas ICRP predicts greater alveolar deposition for larger particles, resulting in a higher total LDSA_*AL*_. This pattern is consistent with previous model comparisons reported by Park et al.^64^

#### 4.1.2 Parametrisation of Nasal Deposition for the Olfactory Pathway

As a sensitivity analysis, the nasal deposition fraction was also estimated using the parametrization of Tian et al.^47^ Although both Garcia et al.^21^ and Tian et al.^47^ derived CFD-based parametrizations of total nasal deposition as a function of particle diffusivity and flow rate, they differ in their methodology, anatomical configuration, and flow conditions. The resting adult inhalation flow rate considered here (15 L min^–1^) corresponds to the upper and lower bounds of the ranges investigated by Garcia et al.^21^ and Tian et al.,^47^ respectively. As shown in Figure 1, using the Tian et al.^47^ parametrization leads to almost two times less UFP olfactory deposition of on average.

#### 4.1.3 Implication for Brain Access

Using ICRP rather than MPPD deposition fractions does not affect the relative importance of the alveolar and olfactory pathways for brain exposure, as alveolar UFP deposition is comparable between the two models. In contrast, replacing the Garcia et al.^21^ nasal deposition parametrization with that of Tian et al.^47^ reduces the relative efficiency of the olfactory pathway by about a factor of 2, while still maintaining it as the dominant pathway (see Supporting Information).

### 4.2 Toxicity Weighting LDSA

Toxicological weighting further increases the relative importance of UFPs, as black carbon and organic compounds, both enriched in the ultrafine size range, receive higher weights. As a result, the UFP contribution to LDSA_AL_^tox^ exceeds that to LDSA_AL_ by several percentage points. Furthermore, neglecting hygroscopic water uptake would also lead to an overestimation of the contribution of ultrafine particles to alveolar deposition, indicating that the relative importance of UFPs is likely conservative in the present analysis.

The contribution of wood and traffic to LDSA_AL_^tox^ and LDSA_Olf_^tox^ exceeds that to LDSA_AL_ and LDSA_Olf_ by a few percent, reflecting the higher toxicological weighting assigned to black carbon and organic compounds that are more abundant in wood heating and traffic emissions than in other sources (see the Supporting Information).

### 4.3 Sensitivity to Translocation Rates

The calculation of the daily dose reaching the olfactory bulb via the bloodstream at PA01H was estimated assuming a translocation rate corresponding to an intact epithelial barrier. The dose reaching the brain by the olfactory pathway was higher by a factor about 1200 than by the bloodstream pathway. This ratio between the dose reaching the brain by the olfactory and the bloodstream is also evaluated assuming a pathologically weakened barrier, i.e., a translocation rate about 10 times higher, as well as for three-year-old children, and using the dry LDSAs rather than the wet LDSAs (see Supporting Information). In all cases, the olfactory pathway dominates over the alveolar pathway. The ratio between the two is the lowest at about 57 when considering a weakened epithelial barrier and when computing the LDSA using the ICRP and Tian et al.^47^ deposition fractions.

Beyond pulmonary exposure, the results highlight the potential importance of olfactory deposition as a pathway for brain-relevant particle dose. Elevated olfactory-deposited surface area (LDSA_Olf_) is observed in urban centers with strong traffic influence, supporting a plausible mechanistic link between traffic-related ultrafine particles and neurological effects reported in epidemiological studies. Although health outcomes are not assessed directly, olfactory deposition is estimated to exceed blood-borne translocation under the tested assumptions, suggesting that neuronal pathways could represent an important route for brain exposure to airborne ultrafine particles. This conclusion remains dependent on assumed olfactory-to-bulb and blood-to-brain translocation fractions. Accounting for additional neuronal pathways, such as the trigeminal pathway, could further increase estimates of upper respiratory tract and brain-relevant particle deposition.

## Supplementary Material



## Notes

Methodology about the calculation of LDSA: calculation of deposition fraction (Text S1); calculation of particle diameters (Text S2); calculation of toxicologically weighted LDSA (Text S3); calculation of dose and dose per unit surface area (Text S4); and systemic and brain dose estimation (Text S5). Model configuration and evaluation: model configuration (Text S6); model to measurement evaluation of pollutant concentrations (Text S7); model to measurement evaluation of LDSA (Text S8); spatial variations and correlations between indicators (Text S9); functional residual capacity, volume of the upper respiratory tract, tidal volume and breathing frequency (Table S1); weight of the particle compound category according to their toxicity (Table S2); model to data comparison of pollutant daily concentrations in different regions during winter 2020/2021 (Table S3); model to measurement comparisons of LDSA_AL_ and LDSA_Olf_ (Table S4); Pearson correlations between PM_2.5_, PNC, LDSA_AL_, LDSA_AL_^tox^, LDSA_Olf_, and LDSA_Olf_^tox^ over Europe and over Greater Paris (Table S5); ratio between the dose reaching the brain by the olfactory and the bloodstream for intact and weakened epithelial barriers at PA01H (Table S6); fate of particles in the human respiratory tract (Figure S1); deposition fractions from the MPPD model and from^21^ for olfactory deposition (Figure S2); maps of PM_2.5_ and PN concentrations over France, Île-de-France, and Paris (Figure S3); stations available for model to data comparisons (Figure S4); model to measurement comparison of the size distribution of the number concentration and of the LDSA (Figure S5); scatter plot of daily observed and simulated LDSA_AL_ and LDSA_Olf_, daily observed and simulated LDSA_Al_ and LDSA_Olf_ (Figure S6); variability of particle indicators across domains (Figure S8); maps of relative differences between LDSA_AL_ humid and dry and LDSA_Olf_ humid and dry (Figure S7); maps of ratio of LDSA_AL_ for three-year-old children versus adults (Figure S9); average contribution of ultrafine particles and of particles of diameters between 100 and 385 nm and larger particles to LDSA_AL_^tox^ (adult), LDSA_AL_^tox^ (3-year-old children) over Western Europe, France, Paris background and Paris streets (Figure S10); maps of contribution of ultrafine particles to LDSA_AL_, PM_2.5_, and PNC over France, Île-de-France, and Paris (Figure S11); maps of contribution of particles of diameters lower than 385 nm to LDSA_AL_, PM_2.5_, and PNC over France, Île-de-France, and Paris (Figure S12); average contribution of wood heating and road traffic to LDSA_AL_^tox^ and LDSA_Olf_^tox^ over France, Paris background and Paris streets (Figure S13); maps of contribution of wood heating to LDSA_AL_ and LDSA_Olf_ over France, Île-de-France, and Paris (Figure S14); maps of contribution of traffic to LDSA_AL_ and LDSA_Olf_ over France, Île-de-France, and Paris (Figure S15); and maps of ratio of the dose reaching the olfactory bulb by the olfactory route to the dose reaching the brain by the systemic circulation (Figure S16) (PDF)
