# Supplementary material for: Quantifying Olfactory and Alveolar Deposition of Ultrafine Particles Using Multiscale Modeling: Implications for Brain Exposure
Source: Environ Sci Technol. 2026 Jun 25;60(27):19465–75. doi: 10.1021/acs.est.6c05750 (PMC13374090; doi:10.1021/acs.est.6c05750)
Supplement: Supplementary file 1 [file es6c05750_si_001.pdf]

Supporting information for "Quantifying olfactory and alveolar deposition of ultrafine particles using multi-scale modeling: implications for brain exposure"

Karine Sartelet[1,*], Lya Lugon[1], Soo-jin Park[1], and François Gaie-Levrel[2]

[1]CEREA, ENPC, Institut Polytechnique de Paris, EDF R&D, IPSL, 77 455 Marne la Vallée, France

[2]AIRPARIF, The air quality observatory for the Paris region, 75 004 Paris, France

[*]Corresponding author: karine.sartelet@enpc.fr

Number of pages: 41

Number of figures: 16

Number of tables: 6

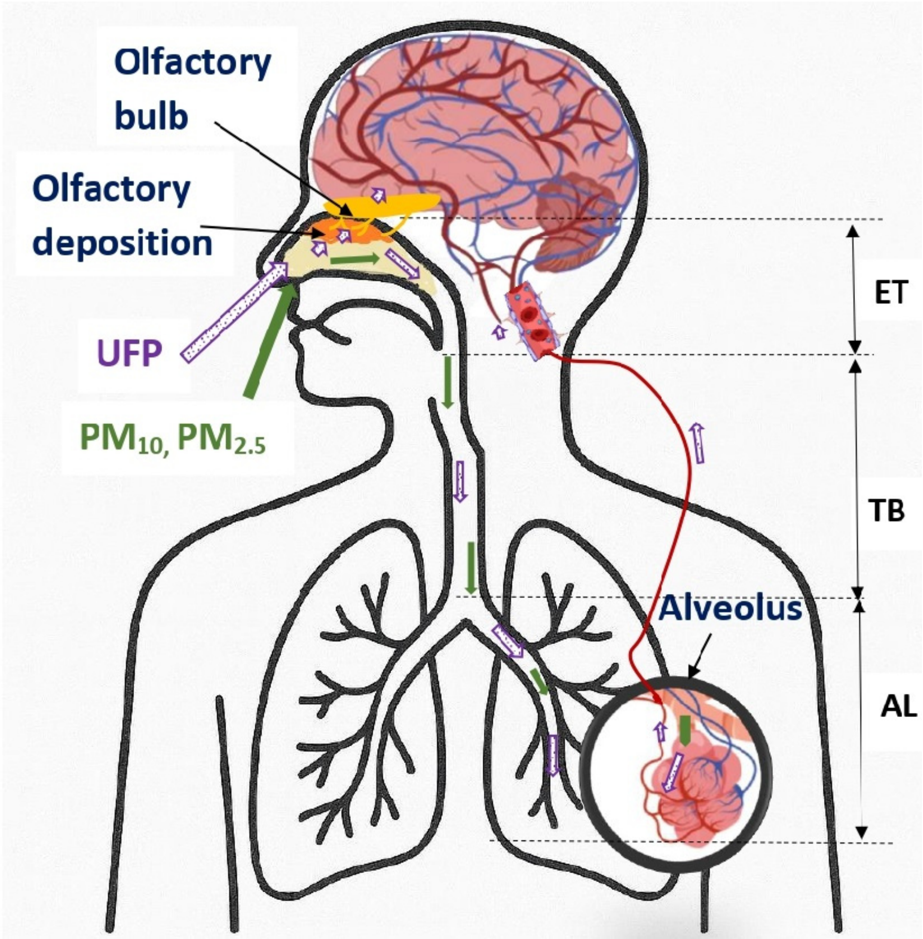

Figure S1: Fate of particles in the human respiratory tract.

S1 Calculation of deposition fraction

With the MPPD model, the particle lung deposition function F_r depends on parameters described in Table S1.

Table S1: Functional Residual Capacity (FRC) (cm^3), Volume of the upper respiratory tract (URT) (in cm^3), tidal volume (cm^3) and breathing frequency (per minute)

	URT	FRC	Tidal volume	Breathing frequency
Adult	3300	50	625	12
3 years old kid	57.46	9.47	121.3	24

The deposition fractions, $F_{nasal,i}$ and $F_{0,i}$, are respectively the fraction of the particles depositing in the nasal region and the fraction of those particles that subsequently deposit in the olfactory region. They are estimated using the curved fitted from computational fluid dynamics simulations by:[1]

$$F_{0,i} = a\ exp\left(-b\frac{D_{p,i}^{c}}{Q^{d}}\right) \quad (1)$$

$$F_{nasal,i} = 1 - exp\left(-a_2\frac{D_{p,i}^{b_2}}{Q^{c_2}}\right) \quad (2)$$

with a = 0.046, b=19, c=0.42, d=0.60, a_2 = 28.3, b_2 = 0.659, c_2 = 0.502 fitted parameters, $D_{p,i}$ the particle diffusivity (in cm^2/s) of particles of aerodynamic

diameter $d_{p,i}$, Q the airflow rate (in L/min). The particle diffusivity is [cm^2/s]

$$D_{p,i} = 10^4 \frac{k_B T \, C_c}{3\pi\mu d_{p,i}} \quad (3)$$

with μ the air dynamic viscosity ($\mu = 1.91 \times 10^{-5}$ [Pa.s] in the body), k_B the Boltzmann constant (1.38×10^{-23} [J/K], T the body temperature [K], $d_{p,i}$ the particle diameter [m], C_c: Cunningham slip correction factor (dimensionless)[2]:

$$Cc = 1 + 2\frac{\lambda}{d_p}\left[1.257 + 0.4exp\left(-1.1\frac{d_p}{\lambda}\right)\right]. \quad (4)$$

where the air mean free path λ is about 0.0692 μm at the body temperature (309 K).

As a sensitivity study, the nasal deposition fraction is also estimated using the parameterization of:[3]

$$F_{nasal,i} = 1 - 1.0013 \; exp\left(-17.87\frac{D^{0.589}}{Q^{0.494}}\right) \quad (5)$$

with D in m^2 s−1 and Q in m^3 s^{-1}.

For a 3-year-old child, the olfactory deposition fraction ($LDSA_{olf}$) is estimated using a representative airflow rate of 5.8 L min^{-1}. Although this value lies below the lower limit of 15 L min^{-1} for which Eq. (2) was originally fitted, previous work indicates that, for particles in the 10-100 nm size range, deposition fractions are comparable at airflow rates of 5.8 and 15 L min^{-1}[3].

The nasal airway volume of a 3-year-old child is taken to be approximately

24.2% of that of an adult[4], corresponding to a nasal airway surface area of about 40% of the adult value. In addition, the ratio of olfactory epithelium area to total nasal cavity area is reported to be higher in children than in adults by a factor of 1.6[5]. Combining these scaling factors yields an olfactory surface area for a 3-year-old child of approximately 64% of the adult value. This scaling factor is supported by[5], who report that olfactory surface area in 5-year-old children averages 84% that of adults, consistent with a smaller ratio expected for 3-year-old children. While direct measurements in 3-year-olds remain unavailable, these findings support the plausibility of our approach. Because olfactory deposition in the 10-100 nm particle size range scales with olfactory surface area[1], the olfactory LDSA computed using Eq. 2 (main paper) is scaled by a factor of 0.64 for a 3-year-old child. The deposition fraction are shown in Fig. S2.

S2 Calculation of particle diameters

The particle size distribution simulated by the aerosol module is represented using ten sectional bins between 0.01 and 10 μm. Each of this ten bins is divided into 12 bins when the integration over diameter is performed in the LDSA calculation.

LDSA can be inferred from MPSS measurements, in the size range between 10 nm and 385 nm. For measurements, the aerodynamic diameter d_p is calculated from the mobility diameter d_m of the MPSS assuming particles to be spherical and

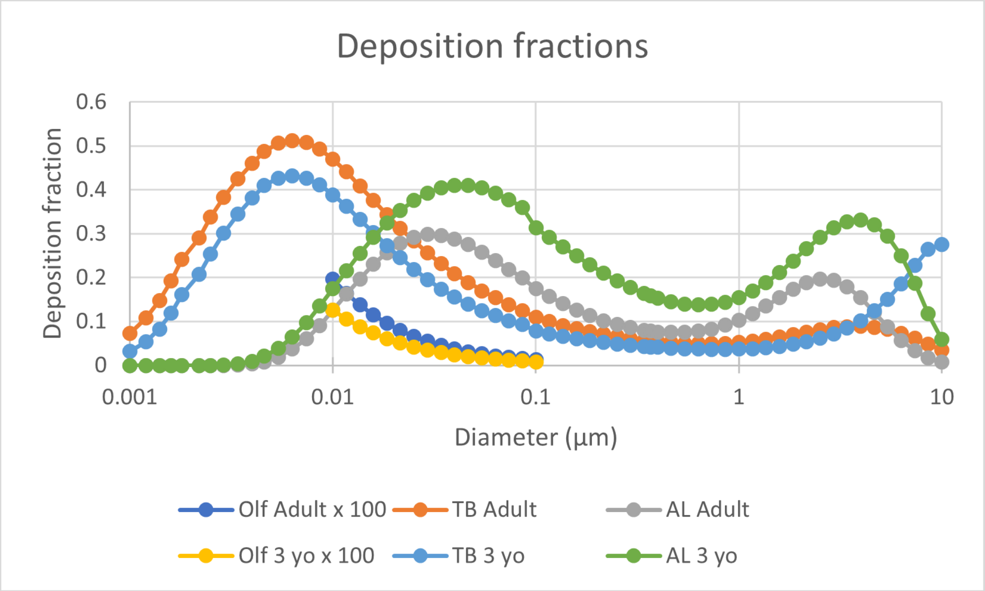

Figure S2: Deposition fractions from the MPPD model (v3.04) and from[1] for olfactory deposition.

with a density ρ_p of 1.3 g cm^{-3} [6]

$$d_p = d_m \sqrt{\frac{\rho_p}{\rho_0}} \sqrt{\frac{C_c(d_m)}{C_c(d_p)}}, \tag{6}$$

with ρ_0 equals 1 g cm^{-3}, and $C_c(d)$ the Cunningham factor for diameter d (see equation (4)). The calculation of d_p from d_m requires a few iterations, as the right hand side of equation (6) also depends on d_p.

For model to measurement comparison, the LDSA is also inferred from the simulated number concentrations using the same methodology as for the measurements, i.e. assuming a dry density of 1.3 g cm^{-3} and diameters d_m corresponding to the dry diameter of particles.

For the different analyses of this paper, except when comparing to measurements, the simulated LDSA takes into account the growth of particles because of the water absorption as they enter the body where the relative humidity can reach 99.5%[7,8]. The absorption of water by particles containing inorganic and hydrophilic organic compounds at ambient relative humidity is calculated during the 3D simulations using the SSH-aerosol software[9] of the multi-scale model chain.

To compute the wet diameter of particles in the body, the growth factor GF, which represents the ratio of particle diameter at a relative humidity RH (d_w) compared to that for dry conditions (d_p) is estimated following[10–12]

$$GF(RH) = \left(1 + \kappa \frac{M_w}{\rho_w} \frac{a_w}{1 - a_w}\right)^{1/3}, \tag{7}$$

$$a_w = \frac{RH}{100\ c_k} \text{ with } c_k = exp\left(\frac{4 M_w \sigma_s}{RT \rho_w d_w}\right) \tag{8}$$

with κ a coefficient from the Köhler theory, M_w and ρ_w the molar mass and density of water, c_k the Kelvin curvature correction factor, σ_s the surface tension (0.072 J/m^2), R the ideal gas constant and T the temperature.

The parameter κ is estimated hourly and for each particle size section from the 3D simulation outputs, from equation (9), rewritten from equation (7):

$$\kappa = \left(GF^3(RH) - 1\right) \frac{\rho_w}{M_w} \frac{1 - a_w}{a_w}, \tag{9}$$

where $GF(RH)$ is the growth factor at ambient RH and T values. It is estimated by the ratio of the simulated wet diameter to dry diameter. Hence, the coefficient

κ differs for each simulation output time and each size section.

The particle growth factor within the respiratory tract (GF_{body}) is calculated using Eq. (7), based on the corresponding κ values and physiological conditions representative of the human body (relative humidity of 99.5% and temperature of 309 K). The numerical evaluation of GF_{body} requires an iterative procedure, as the Kelvin term c_k depends on the resulting wet particle diameter d_w.

Aerodynamic particle diameters within the body are subsequently derived from these wet diameters at 99.5% relative humidity, accounting for particle density and the associated uptake of water:

$$\rho_{body} = \frac{m_{body}}{V_{body}} = \frac{m_p + m_w}{V_{body}} \quad (10)$$

$$\rho_{body} = \frac{\rho_p d_p^3 + \rho_w (d_{body}^3 - d_p^3)}{d_{body}^3} \quad (11)$$

$$\rho_{body} = \rho_w + \frac{\rho_p - \rho_w}{GF_{body}}. \quad (12)$$

with m_p the dry mass, m_w the water mass, m_{body} and V_{body} the mass and volume in the body.

S3 Calculation of toxicologically weighted LDSA

Black carbon and anthropogenic secondary organic aerosols (SOAs) are assigned the highest weights (25% each). Black carbon may contain harmful substances on its surface and a unit of black carbon may be several times more toxic than

a unit of $PM_{2.5}$[13,14]. Anthropogenic secondary organic aerosol may generate reactive oxygen species[15–17]. As aged and oxidized SOA may be more toxic[18,19], anthropogenic SOAs are assigned a higher weight than primary organic aerosols (20%). The weight of biogenic SOA is lower (10%), reflecting generally weaker oxidative potential than anthropogenic SOA[17,18]. Secondary inorganic aerosol (SIA) is given a minimal weight (5%), as species such as sulfate, nitrate, and ammonium may have a minor direct contribution to oxidative potential, although they may modulate it indirectly through effects on solubility[17]. Dust is weighted higher (15%) than inorganics and biogenic organic aerosols[20], due to its content of redox-active metals such as Fe, Mn, and Cu, which contribute to oxidative potential[17]. The toxicity weighted LDSA ($LDSA^{tox}$) is calculated by weighting the LDSA by the mass of the particle compound category using the weights detailed in Table S2.

Table S2: Weight of the particle compound category according to their toxicity. The sum of the weights equals 1.

Category	Weight
Black carbon	0.25
Anthropogenic primary organic aerosol	0.2
Anthropogenic secondary organic aerosol	0.25
Biogenic secondary organic aerosol	0.1
Inorganics	0.05
Dust	0.15

S4 Calculation of dose and dose per unit surface area

For each respiratory region r (AL, Olf), the LDSA concentration $LDSA_r$ [μm^2/cm^3] is converted into an hourly deposited dose D_r [µm^2/h] according to the minute ventilation (VE) as

$$D_r = LDSA_r \times VE \times 60,$$

with the factor 60 converting to an hourly rate, and VE representing the product of tidal volume and breathing frequency (see Table S1).

The hourly LDSA$_r$ dose normalized by body weight is computed by dividing D_r by the body weight. We use 70 kg for adults and 15 kg for a 3-year-old child. Under the same exposure duration, a 3-year-old's dose per kilogram is therefore higher than an adult's by a factor of 1.8 × (LDSA_child / LDSA_adult), that is about 1.8 times the ratio of the LDSA levels.

S5 Systemic and brain dose estimation

For AL, particle kinetics is modeled as a one-compartment system with input the dose D_{AL} (see supporting information) and removal by clearance (k_c), absorption into blood (k_{abs}) and translocation ($k_{transloc}$):

$$\frac{dX_{\mathrm{AL}}}{dt} = D_{\mathrm{AL}} - (k_c + k_{abs} + k_{transloc})X_{\mathrm{AL}}, \tag{13}$$

with $X_{\mathrm{AL}}(0) = 0$ for each independent hour. The hourly translocated dose into blood is then

$$B_{\mathrm{AL}} = \int_0^1 k_{abs}\, X_{\mathrm{AL}}(t)\, dt, \tag{14}$$

which has the closed-form solution

$$B_{\mathrm{AL}} = k_{transloc} \frac{D_{\mathrm{AL}}}{k_c + k_{abs} + k_{transloc}} \left(1 + \frac{e^{-(k_c + k_{abs} + k_{transloc})} - 1}{k_c + k_{abs} + k_{transloc}}\right). \tag{15}$$

For clearance constants k_c, we adopt resting values consistent with ICRP[21]:

$$k_{c,\mathrm{AL}} = \frac{0.0015}{24}\ \mathrm{h}^{-1} \approx 6.25 \times 10^{-5}\ \mathrm{h}^{-1}. \tag{16}$$

We applied ICRP "moderate" absorption kinetics, i.e. a mixture of a fast hydrophilic component ($k_{abs}^{\mathrm{fast}} = 0.125\ \mathrm{h}^{-1}$) and a slow hydrophobic component ($k_{abs}^{\mathrm{slow}} = 2.1 \times 10^{-4}\ \mathrm{h}^{-1}$), assuming equal fractions (50/50).

The translocation kinetic rate may vary with particle size and composition[22–24]. Recent studies suggest that in opposition to fine particles, UFP may not accumulate substantially in the lung[25], which could indicate relatively fast translocation. To assess the sensitivity of brain exposure estimates to alveolar translocation rates, two scenarios are considered: an intact alveolar epithelial barrier, with a translocation rate of $4.2 \times 10^{-4}\ \mathrm{h}^{-1}$ corresponding to approximately 1% translocation over 24 hours as measured in vitro for diesel exhaust particles across alveolar epithelial cells[26], and a pathologically weakened barrier, with a translocation rate of $3 \times 10^{-3}\ \mathrm{h}^{-1}$ corresponding to 7% translocation[26].

The blood dose B over one hour is then equal to B_{AL}. The blood-to-brain translocation fraction, across the very tight blood-brain barrier is very uncertain, and likely to be low[27], even for engineered nanoparticles[28,29]. Here, a fixed fraction $f_{\text{blood}\rightarrow\text{brain}} = 0.001$ of the blood dose is assumed to cross the blood–brain barrier:

$$\text{Brain}_{\text{blood}} = f_{\text{blood}\rightarrow\text{brain}}\, B. \tag{17}$$

UFP can be translocated to the olfactory bulb with studies reporting values of transported particles ranging between 9 and 20% for rodents[30–32]. In humans, this fraction may be lower due to anatomical differences (e.g., thicker olfactory epithelium, longer axonal pathways), but it may align with rodent studies for soluble compounds[33]. Hence, we assume a fraction $f_{\text{Olf}\rightarrow\text{bulb}} = 0.15$ of olfactory deposition reaches the olfactory bulb, but also consider a ten times lower fraction in the sensitivity analysis.

S6 Model configuration

Boundary conditions for the outer domain (FRA9) are taken from Copernicus Atmosphere Monitoring Service (CAMS) reanalyses. Anthropogenic emissions over Europe are derived from the EMEP (European Monitoring and Evaluation Programme) inventory, with a spatial resolution of 0.1° × 0.1°. For simulations over Île-de-France (IDF1 domain), anthropogenic emissions are taken from the bottom-up Airparif inventories. To characterize the use of supplementary and recreational heating, the types of equipment employed, and the

associated wood consumption, Airparif relied on insights from sociological surveys[34]. Traffic emissions are calculated using the HEAVEN traffic emission model (https://www.airparif.asso.fr/heaven-emissions-du-trafic-en-temps-reel, last access: 17 March 2025), with emission fields adjusted using measured traffic count data to better represent the spatial and temporal variability of road traffic emissions. Ultrafine particle emissions are estimated by activity sector using $PM_{0.1}/PM_1$ and $PM_1/PM_{2.5}$ mass ratios, combined with sector-specific particle size distributions prescribed within each size range. This approach follows the methodology described in.[35–37] Overall, as detailed below, simulated concentrations show good agreement with observations for NO_2, PM_{10}, and $PM_{2.5}$, particle number concentration (PNC), and PM chemical composition, including black carbon as well as organic and inorganic components.

S7 Model to measurement evaluation of pollutant concentrations

The concentrations of $PM_{2.5}$ and PNC are shown in Fig. S3.

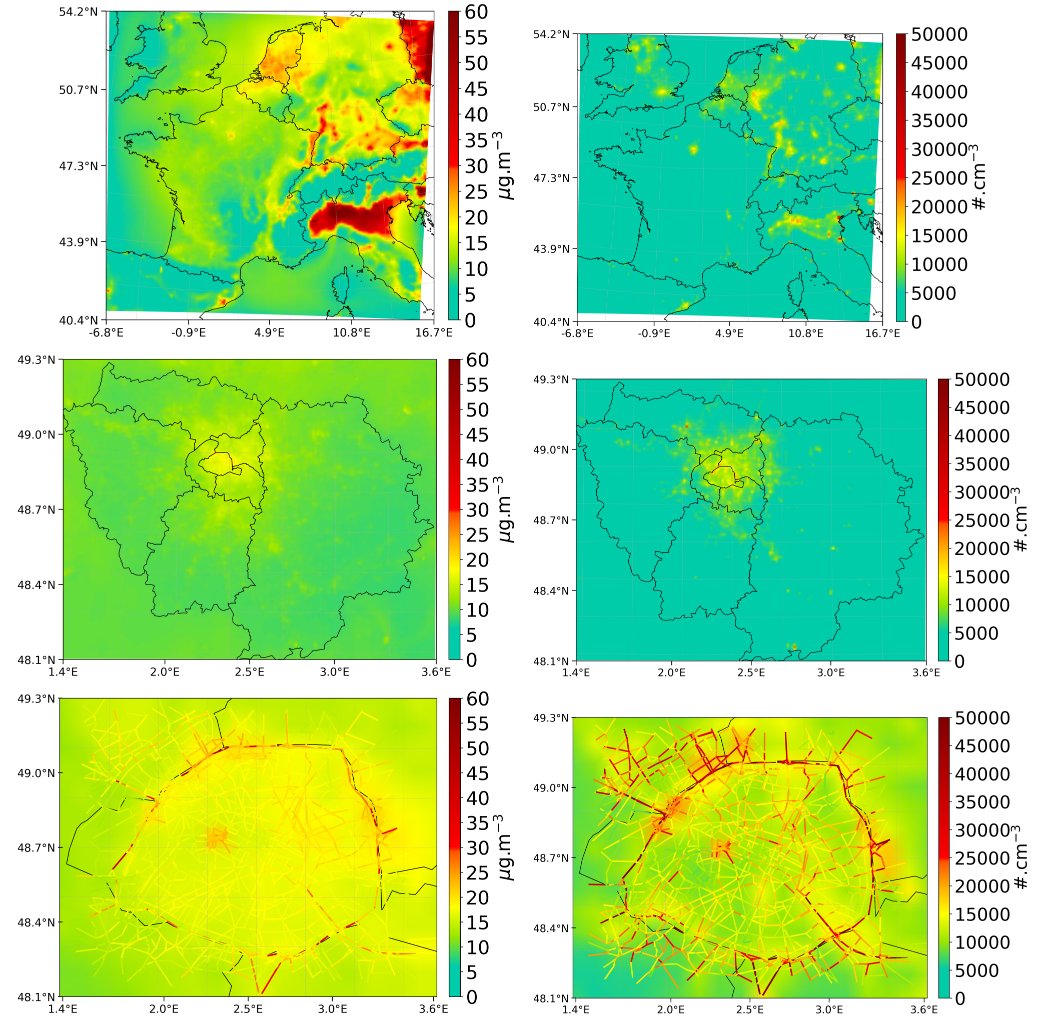

Figure S3: Maps of $PM_{2.5}$ (left panels) and PN (right panels) concentrations over France (top panels), Île-de-France (middle panels) and Paris (bottom panels).

Simulated concentrations of NO_2, PM_{10}, and $PM_{2.5}$ are evaluated against observations from the Geod'air database (https://www.geodair.fr, last access: 17 January 2026), which provides nationwide coverage across France. Simulated par-

ticle chemical composition and size-resolved concentrations are evaluated against measurements conducted at Airparif monitoring stations. Performance statistics for particle number concentration (PNC), $PM_{2.5}$, PM_{10}, and PM chemical composition, including black carbon as well as organic and inorganic components, are reported in Table S3. The spatial distribution of the monitoring stations is shown in Fig. S4.

Overall, simulated concentrations show good agreement with observations. For all pollutants, more than 50% of model predictions fall within a factor of two of the measurements, satisfying the most stringent performance criterion defined by Hanna and Chang [38]. Furthermore, over Île-de-France, the performance criteria proposed by Emery et al. [39] are met for all pollutants (normalized mean error < 50%, normalized mean bias within ±35%, and correlation coefficient > 0.5).

For pollutants with strong local variability, including NO_2, black carbon, and particle number concentration, model performance improves when using the higher-resolution simulation (1 km × 1 km) compared to the larger-scale simulation (9 km × 9 km), highlighting the benefit of fine spatial resolution for capturing localized emission influences. For example for number concentrations, the bias NMB decreases from 52% with a 9 km × 9 km resolution to 0% with a 1 km × 1 km resolution, and the fraction of predictions within a factor 2 (FAC2) increases from 65% to 87%.

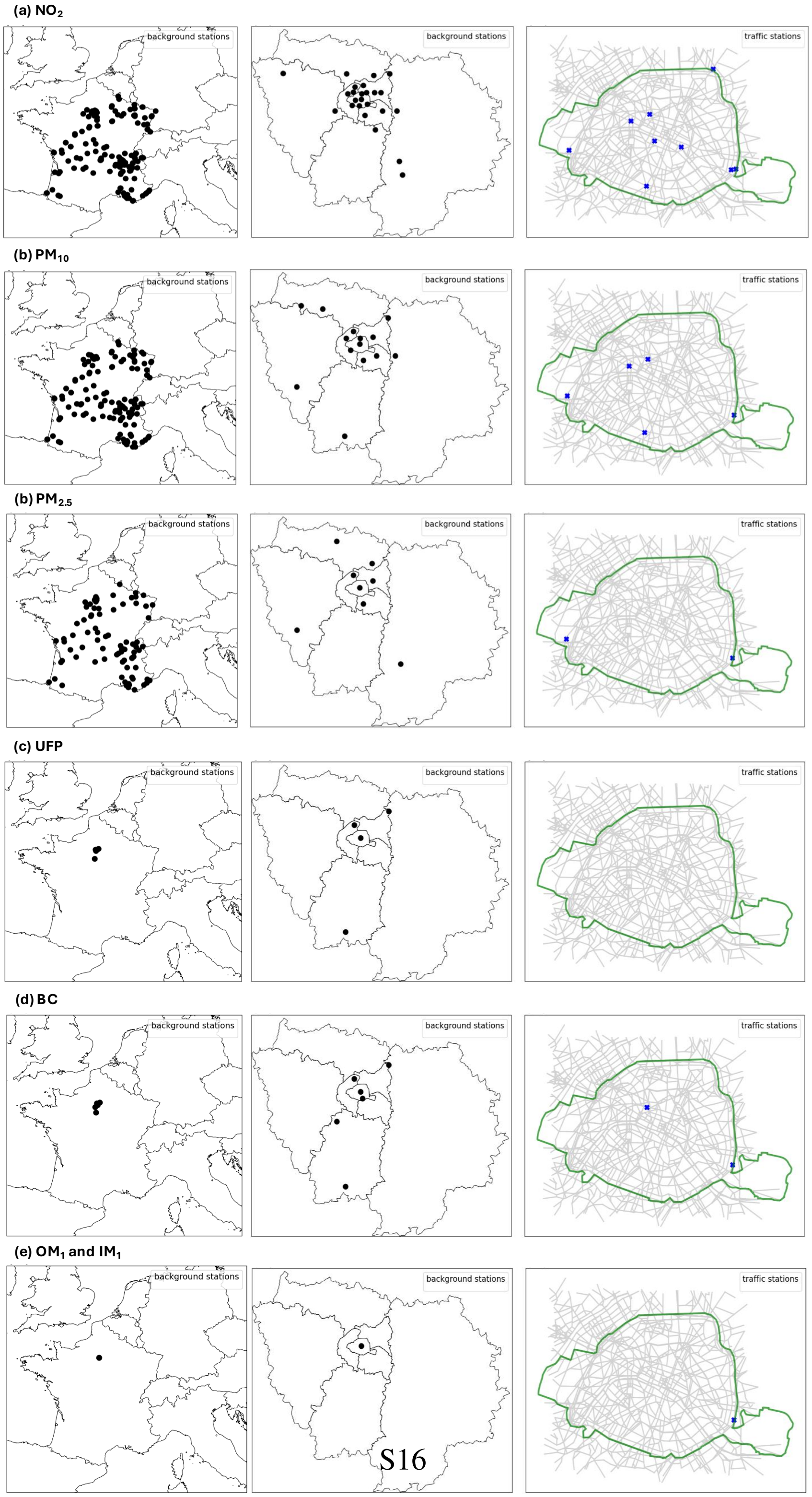

Figure S4: Stations available for model to data comparisons over France (left panels), Île-de-France (middle panels) and Paris streets (right panels). Note that some stations over France may also appear in the Île-de-France panel due to overlapping zoom areas.

Table S3: Model to data comparison of pollutant daily concentrations in different regions during winter 2020/2021. Observed concentrations (Obs.), simulated concentrations (Sim.) and RMSE are expressed in μg m^{-3} (except for PNC for which they are expressed in cm^{-3}), while NMB, NME and FAC2 are in %.

Metrics	Region	Nstat	Obs.	Sim.	RMSE	Corr.	NMB	NME	FAC2
NO_2	France	158	17.1	11.3	10.6	52.4	-35.8	52.6	52.6
	Île-de-France	24	24.9	25.0	8.9	62.3	-0.2	29.6	92.7
	Paris streets	9	51.8	43.1	15.9	48.5	21.7	31.1	96.7
PM_{10}	France	139	16.3	14.1	11.7	51.3	-10.5	46.2	75.6
	Île-de-France	14	16.7	14.6	8.6	57.8	-11.9	33.4	86.2
	Paris streets	5	26.1	23.1	11.1	52.4	-12.0	30.0	92.0
$PM_{2.5}$	France	86	11.2	12.6	7.6	59.7	16.5	52.2	72.5
	Île-de-France	8	11.9	12.6	5.7	64.1	6.1	36.8	86.3
	Paris streets	2	16.5	22.8	8.7	71.4	38.8	44.9	87.3
Number	France	4	7086	10738	6730	43.8	52.2	76.2	64.8
	Île-de-France	4	7086	7078	3196	57.2	0.2	34.5	88.6
	Paris streets	0	n/a	n/a	n/a	n/a	n/a	n/a	n/a
BC	France	6	0.6	1.0	0.8	47.3	81.1	99.2	54.5
	Île-de-France	6	0.6	0.6	0.4	52.0	0.7	43.8	79.8
	Paris streets	2	3.2	3.5	1.6	51.0	26.8	50.1	82.5
OM_1	France	1	3.8	5.5	3.9	51.7	45.1	68.7	75.0
	Île-de-France	1	3.8	4.0	1.6	71.1	3.9	30.1	95.5

	Paris streets	1	4.0	6.6	3.6	57.6	63.0	72.0	68.2
IM_1	France	1	3.7	4.5	2.8	68.4	19.8	55.4	56.8
	Île-de-France	1	3.7	3.6	2.2	80.3	38.4	38.4	80.7
	Paris streets	1	3.9	5.0	2.9	73.6	28.9	55.4	61.4

S8 Model to measurement evaluation of LDSA

The model to measurement statistics averaged over the 4 measurements stations are shown in Table S4. LDSA metrics are inherently more challenging to simulate than mass-based metrics such as $PM_{2.5}$. Estimating deposited surface area requires accurately representing the particle size distribution, particularly in the ultrafine range where both particle surface area and deposition efficiencies vary strongly with diameter. Consequently, any error in the simulated size distribution of particles can translate into large deviations in deposited surface area metrics.

Although no criteria exists for LDSA, following the criteria of[39] for $PM_{2.5}$ (performance goals: normalized mean bias (NMB) within ±10–15%, normalized mean error (NME) $\leq$ 35%, and correlation coefficients (R) $\geq$ 0.70; more relaxed criteria of acceptable performance: ±30–35% for NMB, $\leq$ 50% for NME, and $R \geq 0.40$), both $LDSA_{AL}$ and $LDSA_{Olf}$ compare well to measurements, with a NME of 22-26%, a NMB of -10% and -21%, and a correlation between 68-77%. The fraction of predictions within a factor of two (FAC2) is 96-98%. For FAC2, a strict performance criterion for models is FAC2 greater than 50%[38]. Scatter plots

of the measured to modeled $LDSA_{AL}$ and $LDSA_{Olf}$ and their daily variability, as well as comparisons of the number size distribution and LDSA at the different stations are provided in Fig.S6. The model slightly underestimates LDSA in the ultrafine range at the measurement stations, with normalized mean biases of -21% for $LDSA_{AL}$ and -14% for $LDSA_{Olf}$. This may lead to a modest underestimation of absolute deposition, but is not expected to substantially affect the relative comparison between olfactory and alveolar brain-access pathways because both are affected in the same size range.

Table S4: Model to measurement comparisons of $LDSA_{AL}$ and $LDSA_{Olf}$ including mean observed (Obs.) and simulated (Sim.) values, root mean square error (RMSE), correlation coefficient (Corr.), normalized mean bias (NMB), normalized mean error (NME), and fraction of predictions within a factor of two (FAC2), averaged over all stations.

Metric	Obs.	Sim.	rmse	Corr.	NMB	NME	FAC2
	$\mu m^2\ cm^{-3}$	$\mu m^2\ cm^{-3}$	$\mu m^2\ cm^{-3}$	%	%	%	%
$LDSA_{AL}$	16.7	15.1	6.5	77	-10	25	98
$LDSA_{AL,\text{ultrafine}}$	7.7	6.0	2.9	72	-21	26	96
$LDSA_{Olf}$	0.0101	0.0086	0.003	68	-14	22	98

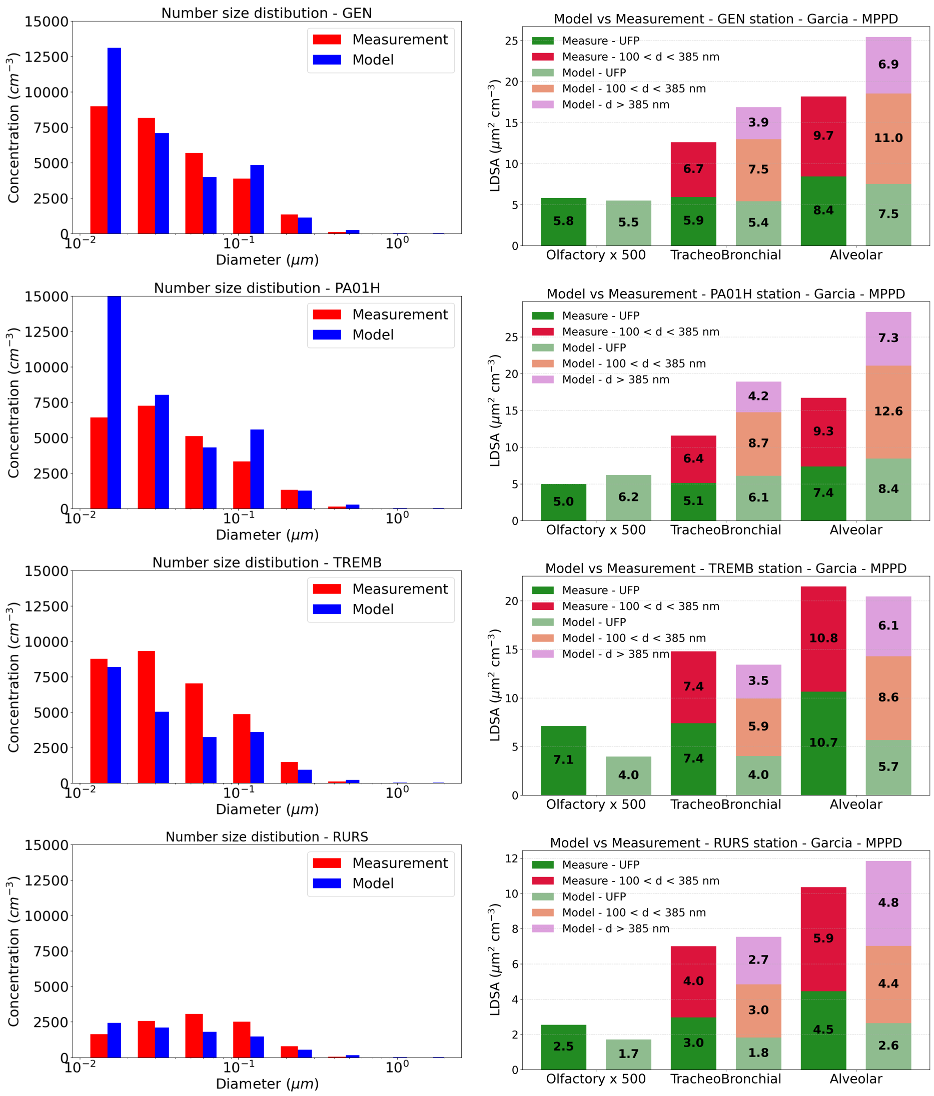

Figure S5: Model to measurement comparison of the size distribution of the number concentration (left column) and of the LDSA (right column) at the four measurement stations (GEN on the first line, PA01H on the second line, TREMB on the third line, RURS on the fourth line).

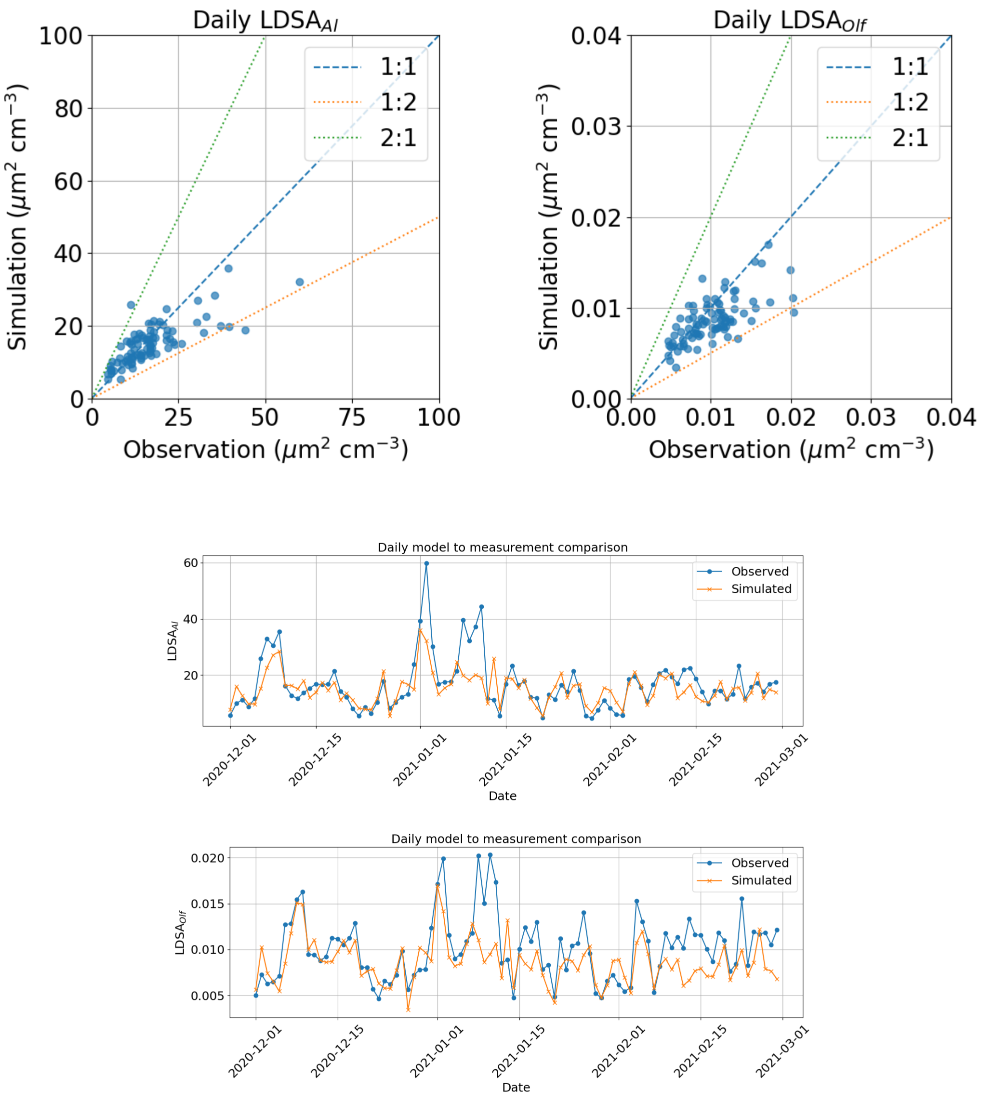

Figure S6: Top panels: scatter plot of daily observed and simulated LDSA$_{AL}$ (left panel) and LDSA$_{Olf}$ (right panel) at the different stations. The dashed line is 1:1 (perfect agreement). Dotted lines indicate the FAC2 bounds (2:1 and 1:2). Points between the dotted lines are within a factor of two. Middle (and bottom) panels: daily observed and simulated LDSA$_{Al}$ (and LDSA$_{Olf}$ resp.) averaged over the stations for the simulation period.

S9 Spatial variations and correlations between indicators

Quantitative analyses indicate that surface-based exposure metrics vary more strongly in space than particle mass, particularly at fine spatial scales. Coefficients of variation (CVs) across metrics and spatial scales are shown in Fig. S7. At urban to national scales (Paris, Île-de-France, and France), variability is lower for $PM_{2.5}$ (Paris and Île-de-France: ≈ 0.16; France: ≈ 0.33) than for particle number concentration (PNC; $\approx$ 0.47–0.50 across scales).

Accounting for particle-bound water (i.e., humid rather than dry LDSA) increases the spatial variability of LDSA metrics, with LDSA_{Olf} systematically higher than LDSA_{Olf}^{dry} and LDSA_{AL} higher than LDSA_{AL}^{dry}. This reflects hygroscopic growth during atmospheric ageing, which modifies particle size, diffusivity, and regional deposition efficiencies. When hygroscopic growth is not considered, alveolar deposition of ultrafine particles is reduced and spatial contrasts are slightly smoothed.

LDSA metrics are systematically more variable than mass-based metrics, with LDSA_{Olf} exhibiting particularly large CVs (Paris: ≈ 0.66; Île-de-France: ≈ 0.87; France: ≈ 0.74), exceeding those of PNC. Toxicologically weighted LDSA metrics show the largest variability (e.g., $\text{LDSA}_{Olf}^{tox} \approx 0.70$ over Paris and 0.80 over France), as relatively homogeneous secondary inorganic components receive lower weight. Strikingly, variability in LDSA_{Olf} among individual streets within Paris approaches that observed across France as a whole, highlighting the magnitude of

local exposure inequalities within cities.

Across Western Europe, $PM_{2.5}$ and PNC display comparable variability ($\approx$ 1.1–1.2), likely reflecting the coarse 9 km resolution, which smooths spatial contrasts in number concentrations. In contrast, toxicity-weighted LDSA metrics exhibit substantially higher variability ($\approx$ 1.5–1.7).

Correlation coefficients between $PM_{2.5}$, PNC, and LDSA metrics are reported in Table S5. $PM_{2.5}$ shows strong correlations with $LDSA_{AL}$ (r = 0.94 over Europe; r = 0.89 over Île-de-France) but weak correlations with $LDSA_{Olf}$ (r = 0.43 and r = 0.32, respectively). PNC correlates more strongly with $LDSA_{Olf}$ (r = 0.81 across Europe and r = 0.86 in Paris), consistent with their shared sensitivity to ultrafine particles. Correlations decrease when toxicological weighting is applied. $LDSA_{AL}$ and $LDSA_{Olf}$ are only moderately correlated (r = 0.53 in Europe and r = 0.59 in Paris), reflecting their overlapping but distinct size sensitivities. $PM_{2.5}$ and $LDSA_{AL}$ showed a strong spatial correlation over the full domains, mainly driven by regional-scale background patterns. Yet, important differences emerge in urban areas, where ultrafine particles can strongly enhance deposited surface area without substantially affecting $PM_{2.5}$ mass.

Table S5: Pearson correlations between $PM_{2.5}$, PNC, $LDSA_{AL}$, $LDSA_{AL}^{tox}$, $LDSA_{Olf}$ and $LDSA_{Olf}^{tox}$ over Europe under the diagonal and over Greater Paris above.

	$PM_{2.5}$	PNC	$LDSA_{AL}$	$LDSA_{AL}^{tox}$	$LDSA_{Olf}$	$LDSA_{Olf}^{tox}$
$PM_{2.5}$	1	0.59	0.89	0.71	0.32	0.29
PNC	0.64	1	0.82	0.82	0.86	0.82
$LDSA_{AL}$	0.94	0.72	1	0.92	0.59	0.62
$LDSA_{AL}^{tox}$	0.87	0.63	0.97	1	0.74	0.75
$LDSA_{Olf}$	0.43	0.81	0.53	0.52	1	0.99
$LDSA_{Olf}^{tox}$	0.43	0.78	0.53	0.53	0.99	1

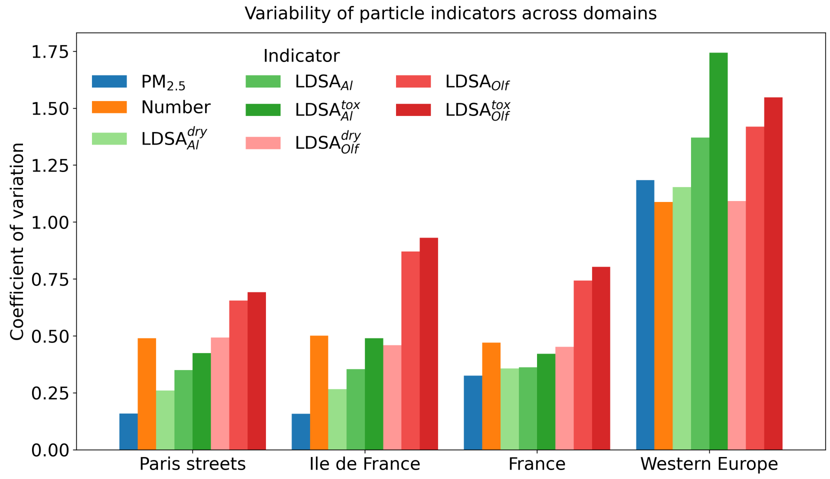

Figure S7: Variability of particle indicators across domains.

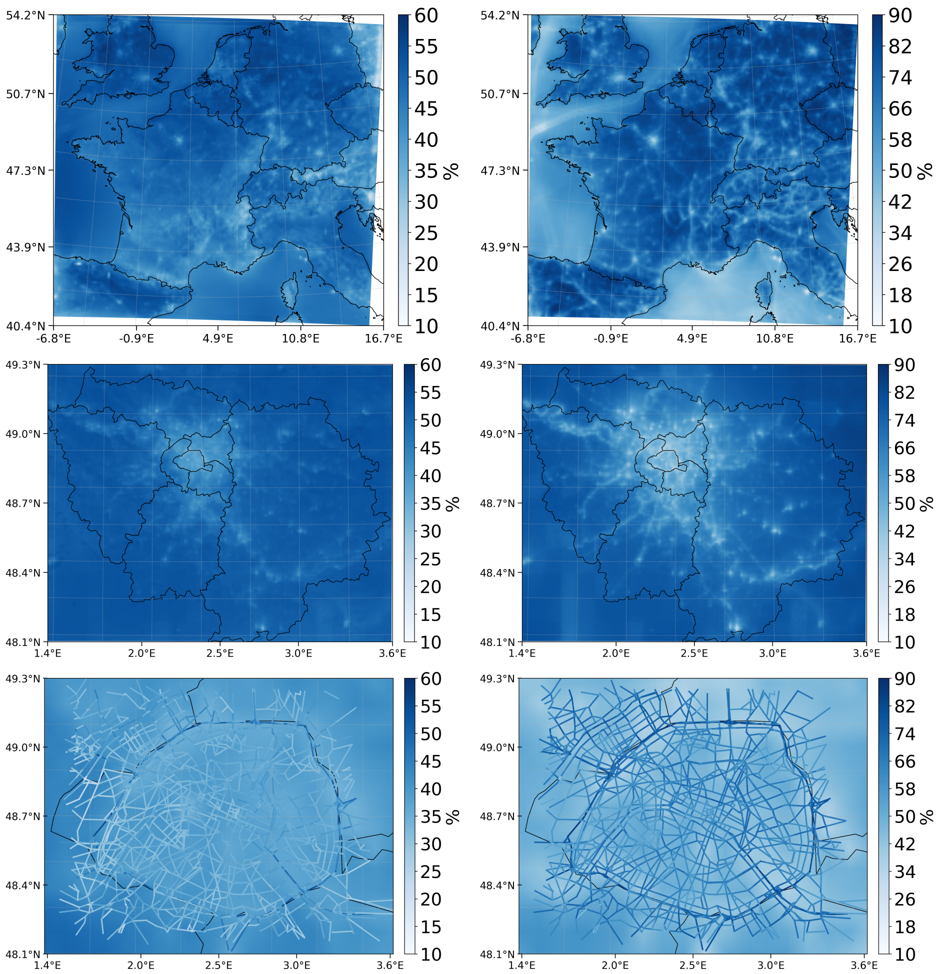

Figure S8: Maps of relative differences between LDSA_{AL} humid and dry (left columns) and LDSA_{Olf} humid and dry (right columns) over France (top panels), Île-de-France (second line panels) and Paris (third line panels). Simulations with and without taking into account the water absorbed by particles in the calculation of the deposition fractions are compared.

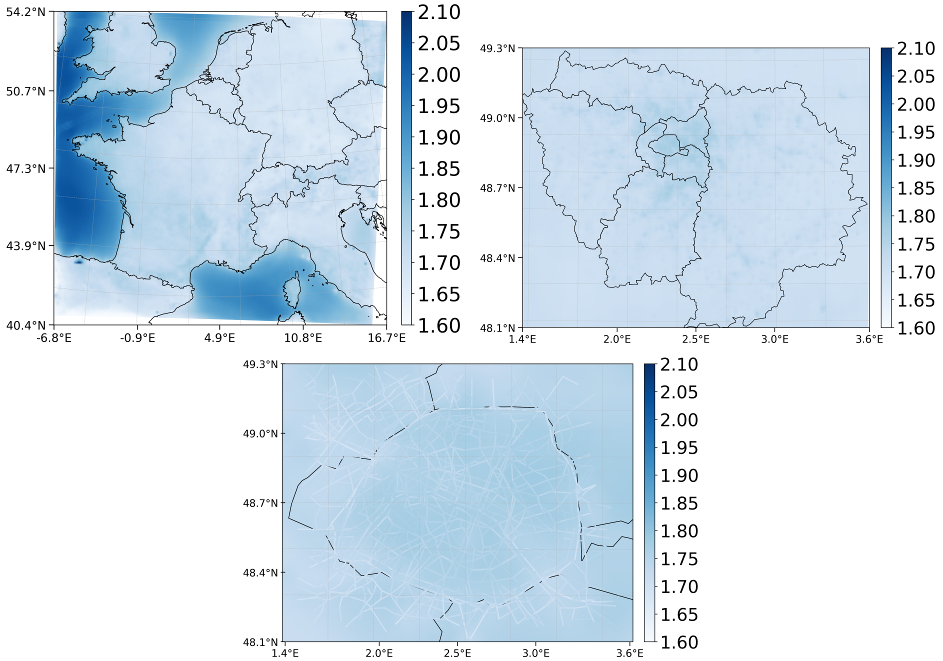

Figure S9: Maps of ratio of $LDSA_{AL}$ for three-year-old children versus adults over France (top left panel), Île-de-France (top right panel) and Paris (bottom panel).

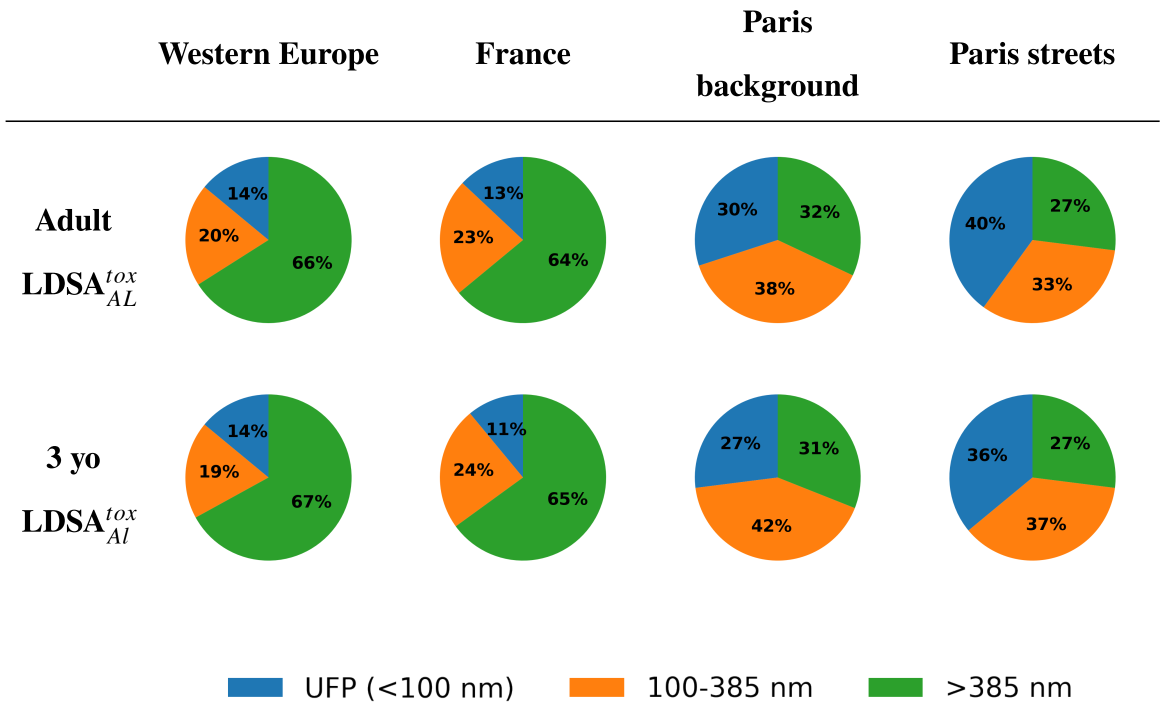

Figure S10: Average contribution of ultrafine particles (in blue) and of particles of diameters between 100 nm and 385 nm (in orange) and larger particles (in green) to LDSA$^{tox}_{AL}$ (adult), LDSA$^{tox}_{AL}$ (3-year-old children) over Western Europe, France, Paris background and Paris streets.

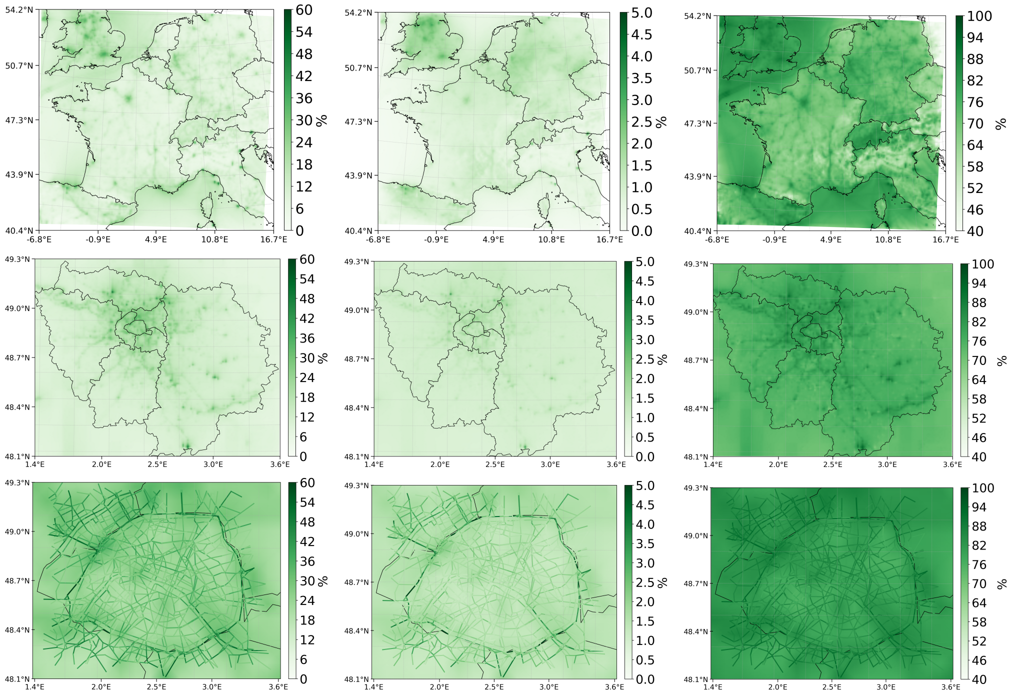

Figure S11: Maps of contribution of ultrafine particles to $LDSA_{AL}$ (left columns), $PM_{2.5}$ (middle columns) and PNC (right columns) over France (top panels), Île-de-France (middle panels) and Paris (bottom panels).

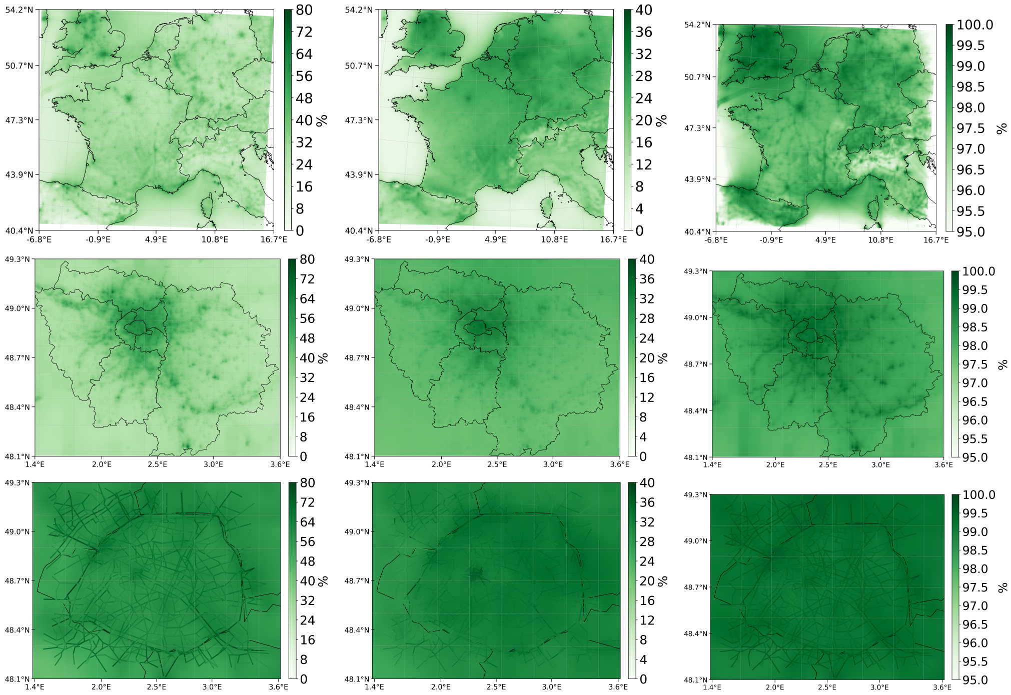

Figure S12: Maps of contribution of particles of diameters lower than 385 nm to $LDSA_{AL}$ (left columns), $PM_{2.5}$ (middle columns) and PNC (right columns) over France (top panels), Île-de-France (middle panels) and Paris (bottom panels).

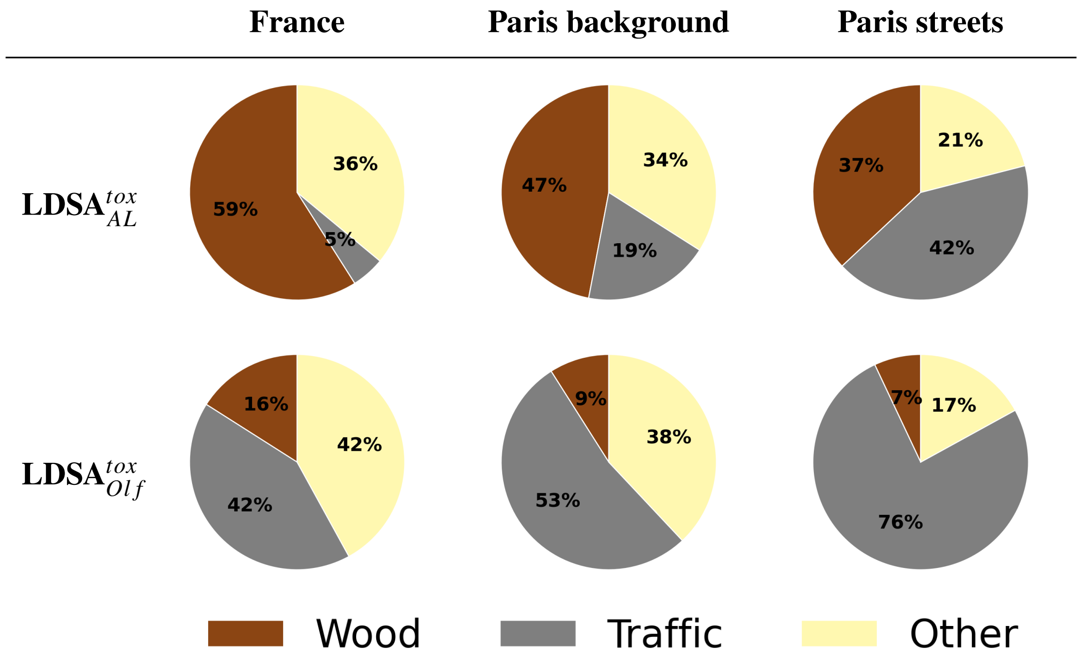

Figure S13: Average contribution of wood heating and road traffic to LDSA$^{tox}_{AL}$ and LDSA$^{tox}_{Olf}$ over France, Paris background and Paris streets. While over France the contribution is linked to nationwide wood heating and traffic, over Paris it is only from regional wood heating and traffic.

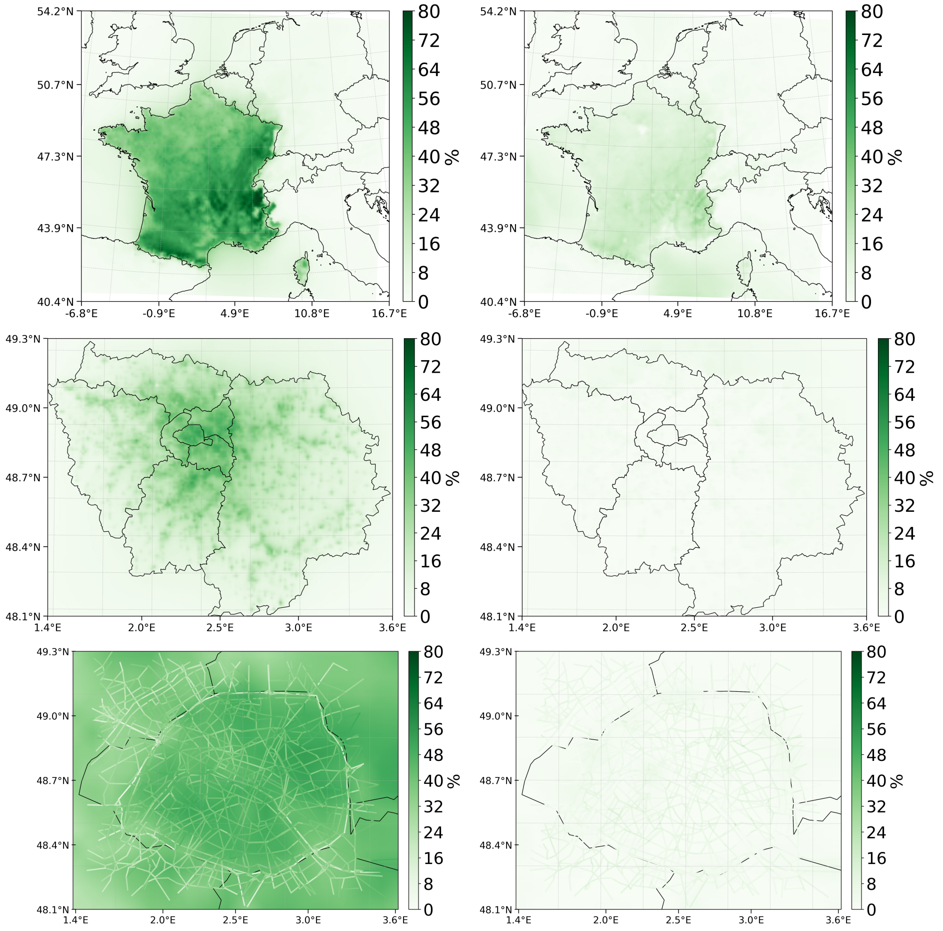

Figure S14: Maps of contribution of wood heating to $LDSA_{AL}$ (left columns) and $LDSA_{Olf}$ (right columns) over France (top panels), Île-de-France (second line panels) and Paris (third line panels). While over France the contribution is linked to nationwide wood heating and traffic, over Paris it is only from regional wood heating and traffic.

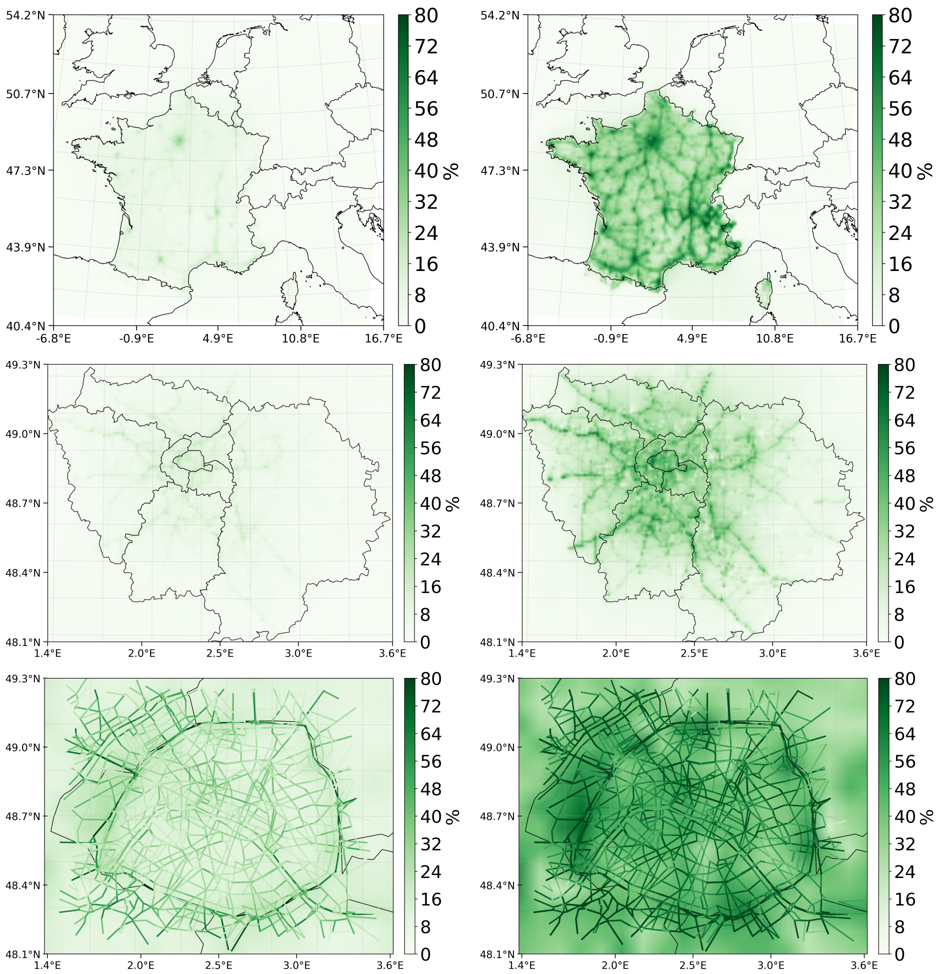

Figure S15: Maps of contribution of traffic to $LDSA_{AL}$ (left columns) and $LDSA_{Olf}$ (right columns) over France (top panels), Île-de-France (second line panels) and Paris (third line panels). While over France the contribution is linked to nationwide wood heating and traffic, over Paris it is only from regional wood heating and traffic.

Table S6: Ratio between the dose reaching the brain by the olfactory and the bloodstream for intact and weakened epithelial barriers at PA01H. Values are dimensionless, as they correspond to ratios between doses expressed in identical units. The calculations are performed for adults, using the MPPD and Garcia et al.[1] deposition fractions for calculation of LDSA and taking into account absorption of humidity. Comparisons are performed for three-year-old children as well as using the ICRP and Tian et al.[3] deposition fractions and ignoring absorption of humidity.

	Intact barrier	Weakened barrier
Adult wet LDSA	1200	168
Adult dry LDSA	1065	149
Three-year old wet LDSA	766	107
Adult wet LDSA (ICRP and Tian)	405	57

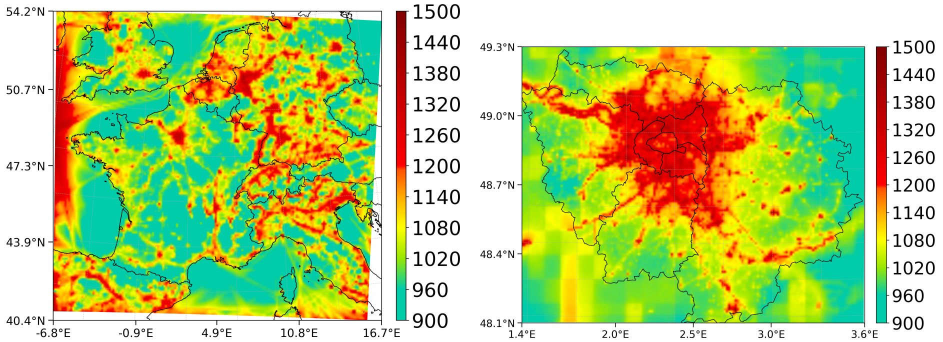

Figure S16: Maps of ratio of the dose reaching the olfactory bulb by the olfactory route to the dose reaching the brain by the systemic circulation. Values are dimensionless, as they correspond to ratios between doses expressed in identical units.

References

[1] Garcia, G. J. M.; Schroeter, J. D.; Kimbell, J. S. Olfactory deposition of inhaled nanoparticles in humans. *Inhal. Toxicol.* **2015**, *27*, 394–403, DOI: 10.3109/08958378.2015.1066904.

[2] Hinds, W. C.; Zhu, Y. *Aerosol technology: properties, behavior, and measurement of airborne particles*; John Wiley & Sons, 2022.

[3] Tian, L.; Shang, Y.; Dong, J.; Inthavong, K.; Tu, J. Human nasal olfactory deposition of inhaled nanoparticles at low to moderate breathing rate. *J. Aerosol Sci.* **2017**, *113*, 189–200, DOI: 10.1016/j.jaerosci.2017.08.006.

[4] Xi, J.; Si, X.; Zhou, Y.; Kim, J.; Berlinski, A. Growth of Nasal and Laryngeal

Airways in Children: Implications in Breathing and Inhaled Aerosol Dynamics. *Respir. Care* **2014**, *59*, 263–273, DOI: 10.4187/respcare.02568.

[5] Sun, Q.; Zhang, Y.; Tian, L.; Tu, J.; Corley, R.; Kuprat, A. P.; Dong, J. Investigation of inter-subject variation in ultrafine particle deposition across human nasal airways: A study involving children, adults, and the elderly. *Sci. Total Environ.* **2024**, *955*, 177028, DOI: 10.1016/j.scitotenv.2024.177028.

[6] DeCarlo, P.; Slowik, J.; Worsnop, D.; Davidovits, P.; Jimenez, J. Particle Morphology and Density Characterization by Combined Mobility and Aerodynamic Diameter Measurements. Part 1: Theory. *Aer. Sci. and Technol.* **2004**, *38*, 1185–1205, DOI: 10.1080/027868290903907.

[7] Sommer, F.; Kroger, R.; Lindemann, J. Numerical simulation of humidification and heating during inspiration within an adult nose. *Rhinology* **2012**, *50*, 157–164, DOI: 10.4193/Rhino11.231.

[8] Shamohammadi, H.; Mehrabi, S.; Sadrizadeh, S.; Yaghoubi, M.; Abouali, O. 3D numerical simulation of hot airflow in the human nasal cavity and trachea. *Comput. Biol. Med.* **2022**, *147*, 105702, DOI: 10.1016/j.compbiomed.2022.105702.

[9] Sartelet, K.; Wang, Z.; Kim, Y.; Lannuque, V.; Couvidat, F. Advanced modeling of gas chemistry and aerosol dynamics with SSH-aerosol v2.0. *Geosci. Model Dev.* **2026**, *19*, 389–421, DOI: 10.5194/gmd-19-389-2026.

[10] Rissler, J.; Svenningsson, B.; Fors, E. O.; Bilde, M.; Swietlicki, E. An evaluation and comparison of cloud condensation nucleus activity models: Predicting particle critical saturation from growth at subsaturation. *J. Geophys. Res. Atmos.* **2010**, *115*, ., DOI: 10.1029/2010JD014391.

[11] Vu, T.; Delgado-Saborit, J.; Harrison, R. A review of hygroscopic growth factors of submicron aerosols from different sources and its implication for calculation of lung deposition efficiency of ambient aerosols. *Air Qual Atmos Health* **2015**, *8*, 429–440, DOI: 10.1007/s11869-015-0365-0.

[12] Ma, L. et al. Deposition potential of 0.003-10μm ambient particles in the humidified human respiratory tract: Contribution of new particle formation events in Beijing. *Ecotoxicol. Environ. Saf.* **2022**, *243*, 114023, DOI: 10.1016/j.ecoenv.2022.114023.

[13] Grahame, T. J.; Klemm, R.; Schlesinger, R. B. Public health and components of particulate matter: The changing assessment of black carbon. *J. Air Waste Manag. Assoc.* **2014**, *64*, 620–660, DOI: 10.1080/10962247.2014.912692.

[14] Niranjan, R.; Thakur, A. The Toxicological Mechanisms of Environmental Soot (Black Carbon) and Carbon Black: Focus on Oxidative Stress and Inflammatory Pathways. *Front. Immunol.* **2017**, *8*, 763, DOI: 10.3389/fimmu.2017.00763.

[15] Tuet, W. Y.; Chen, Y.; Xu, L.; Fok, S.; Gao, D.; Weber, R. J.; Ng, N. L. Chemi-

cal oxidative potential of secondary organic aerosol (SOA) generated from the photooxidation of biogenic and anthropogenic volatile organic compounds. *Atmos. Chem. Phys.* **2017**, *17*, 839–853, DOI: 10.5194/acp-17-839-2017.

[16] Bates, J. T.; Fang, T.; Verma, V.; Zeng, L.; Weber, R. J.; Tolbert, P. E.; Abrams, J. Y.; Sarnat, S. E.; Klein, M.; Mulholland, J. A.; Russell, A. G. Review of Acellular Assays of Ambient Particulate Matter Oxidative Potential: Methods and Relationships with Composition, Sources, and Health Effects. *Environ. Sci. and Technol.* **2019**, *53*, 4003–4019, DOI: 10.1021/acs.est.8b03430.

[17] Daellenbach, K. et al. Sources of particulate-matter air pollution and its oxidative potential in Europe. *Nature* **2020**, *587*, 414–419, DOI: 10.1038/s41586-020-2902-8.

[18] Offer, S. et al. Effect of Atmospheric Aging on Soot Particle Toxicity in Lung Cell Models at the Air-Liquid Interface: Differential Toxicological Impacts of Biogenic and Anthropogenic Secondary Organic Aerosols (SOAs). *Environ. Health Perspect.* **2022**, *130*, 027003, DOI: 10.1289/EHP9413.

[19] Verma, P. K.; Devaprasad, M.; Dave, J.; Meena, R.; Bhowmik, H.; Tripathi, S. N.; Rastogi, N. Summertime oxidative potential of atmospheric PM2.5 over New Delhi: Effect of aerosol ageing. *Sci. Total Environ.* **2024**, *920*, 170984, DOI: 10.1016/j.scitotenv.2024.170984.

[20] Park, M.; Lee, S.; Lee, H.; Denna, M. C. F. J.; Jang, J.; Oh, D.; Bae, M.-S.;

Jang, K.-S.; Park, K. New health index derived from oxidative potential and cell toxicity of fine particulate matter to assess its potential health effect. *Heliyon* **2024**, *10*, e25310, DOI: 10.1016/j.heliyon.2024.e25310.

[21] ICRP Human Respiratory Tract Model for Radiological Protection. *Annals of the ICRP* **1994**, *24*, 1–482, ICRP Publication 66.

[22] Kreyling, W. G.; Semmler, M.; Erbe, F. G.; Mayer, P.; Takenaka, S.; Schulz, H.; Oberdörster, G.; Ziesenis, A. Translocation of ultrafine insoluble iridium particles from lung epithelium to extrapulmonary organs is size dependent but very low. *J Toxicol Environ Health A* **2002**, *65*, 1513–1530, DOI: 10.1080/00984100290071649.

[23] Choi, H. S.; Ashitate, Y.; Lee, J. H.; Kim, S. H.; Matsui, A.; Insin, N.; Bawendi, M. G.; Semmler-Behnke, M.; Frangioni, J. V.; Tsuda, A. Rapid translocation of nanoparticles from the lung airspaces to the body. *Nat. Biotechnol* **2010**, *28*, 1300–1303, DOI: 10.1038/nbt.1696.

[24] Semmler, M.; Seitz, J.; Erbe, F.; Mayer, P.; Heyder, J.; Oberdörster, G.; Kreyling, W. G. Long-term clearance kinetics of inhaled ultrafine insoluble iridium particles from the rat lung, including transient translocation into secondary organs. *Inhal. Toxicol.* **2004**, *16*, 453–459, DOI: 10.1080/08958370490439650.

[25] Kuntic, M. et al. Differential inflammation, oxidative stress and cardiovascular damage markers of nano- and micro-particle exposure in mice: Impli-

cations for human disease burden. *Redox Biology* **2025**, *83*, 103644, DOI: 10.1016/j.redox.2025.103644.

[26] Gunasingam, G.; He, R.; Taladriz-Blanco, P.; Balog, S.; Petri-Fink, A.; Rothen-Rutishauser, B. Combining analytical techniques to assess the translocation of diesel particles across an alveolar tissue barrier in vitro. *Part fibre toxicol.* **2024**, *21*, 1–16, DOI: 10.1186/s12989-024-00585-7.

[27] Qi, Y.; Wei, S.; Xin, T.; Huang, C.; Pu, Y.; Ma, J.; Zhang, C.; Liu, Y.; Lynch, I.; Liu, S. Passage of exogeneous fine particles from the lung into the brain in humans and animals. *Proc. Natl. Acad. Sci.* **2022**, *119*, e2117083119, DOI: 10.1073/pnas.2117083119.

[28] Saraiva, C.; Praça, C.; Ferreira, R.; Santos, T.; Ferreira, L.; Bernardino, L. Nanoparticle-mediated brain drug delivery: Overcoming blood-brain barrier to treat neurodegenerative diseases. *J Control Release* **2016**, *235*, 34–47, DOI: 10.1016/j.jconrel.2016.05.044.

[29] Wehn, A. C.; Krestel, E.; Harapan, B. N.; Klymchenko, A.; Plesnila, N.; Khalin, I. To see or not to see: In vivo nanocarrier detection methods in the brain and their challenges. *J Control Release* **2024**, *371*, 216–236, DOI: 10.1016/j.jconrel.2024.05.044.

[30] Oberdörster, G.; Sharp, Z.; Atudorei, V.; Elder, A.; Gelein, R.; Kreyling, W.; Cox, C. Translocation of inhaled ultrafine particles to the brain. *Inhal. Toxicol.* **2004**, *16*, 437–445, DOI: 10.1080/08958370490439597.

[31] Elder, A.; Gelein, R.; Silva, V.; Feikert, T.; Opanashuk, L.; Carter, J.; Potter, R.; Maynard, A.; Ito, Y.; Finkelstein, J.; Oberdörster, G. Translocation of inhaled ultrafine manganese oxide particles to the central nervous system. *Environ. Health Perspect.* **2006**, *114*, 1172–1178, DOI: 10.1289/ehp.9030.

[32] Patchin, E.; Anderson, D.; Silva, R.; Uyeminami, D.; Scott, G.; Guo, T.; Van Winkle, L.; Pinkerton, K. Size-Dependent Deposition, Translocation, and Microglial Activation of Inhaled Silver Nanoparticles in the Rodent Nose and Brain. *Environ. Health Perspect.* **2016**, *124*, 1870–1875, DOI: 10.1289/EHP234.

[33] Shiga, H.; Taki, J.; Yamada, M.; Washiyama, K.; Amano, R.; Matsuura, Y.; Matsui, O.; Tatsutomi, S.; Yagi, S.; Tsuchida, A.; Yoshizaki, T.; Furukawa, M.; Kinuya, S.; Miwa, T. Evaluation of the Olfactory Nerve Transport Function by SPECT-MRI Fusion Image with Nasal Thallium-201 Administration. *Mol. Imaging Biol.* **2011**, *13*, 1262–1266, DOI: 10.1007/s11307-010-0461-3.

[34] Lugon, L.; Kimmerlin, C.; Kim, Y.; Pousset, P.; Achille, J.; Joly, F.; Couvidat, F.; Collet, S.; Cuniasse, B.; Redaelli, M.; Sartelet, K. Multi-pollutant contribution of wood heating to emissions, concentrations and population exposure during winter time down to the street levels. *J. Hazard. Mater.* **2026**, *506*, 141603, DOI: 10.1016/j.jhazmat.2026.141603.

[35] Sartelet, K.; Kim, Y.; Couvidat, F.; Merkel, M.; Petäjä, T.; Sciare, J.; Wiedensohler, A. Influence of emission size distribution and nucleation on number

concentrations over Greater Paris. *Atmos. Chem. Phys.* **2022**, *22*, 8579–8596, DOI: 10.5194/acp-22-8579-2022.

[36] Park, S. et al. Population exposure to outdoor NO_2, black carbon, and ultrafine and fine particles over Paris with multi-scale modelling down to the street scale. *Atmos. Chem. Phys.* **2025**, *25*, 3363–3387, DOI: 10.5194/acp-25-3363-2025.

[37] Sartelet, K. et al. Air pollution mapping and variability over five European cities. *Environ. Int.* **2025**, *199*, 109474, DOI: 10.1016/j.envint.2025.109474.

[38] Hanna, S. R.; Chang, J. C. Acceptance criteria for urban dispersion model evaluation. *Meteorol. Atmos. Phys.* **2012**, *116*, DOI: 10.1007/s00703-011-0177-1.

[39] Emery, C.; Liu, Z.; Russell, A.; Odman, M. T.; Yarwood, G.; Kumar, N. Recommendations on statistics and benchmarks to assess photochemical model performance. *J. Air Waste Manag. Assoc.* **2017**, *67*, 582–598, DOI: 10.1080/10962247.2016.1265027.
